# Caspase cleavage of Kaposi sarcoma-associated herpesvirus proteins: a role for K5 in preventing caspase-mediated cell death during lytic replication

**DOI:** 10.1128/jvi.00622-25

**Published:** 2025-08-29

**Authors:** David A. Davis, Yana Astter, Emma N. Treco, Prabha Shrestha, Alexandra Stream, Muzammel Haque, Naomi Mulugeta, Robert Yarchoan

**Affiliations:** 1 HIV and AIDS Malignancy Branch, Center for Cancer Research, National Cancer Institute 272101, Bethesda, Maryland, USA; 2 Division of Biotechnology and Molecular Medicine, Department of Pathobiological Sciences, School of Veterinary Medicine, Louisiana State University 5779, Baton Rouge, Louisiana, USA; Lerner Research Institute, Cleveland Clinic, Cleveland, Ohio, USA

**Keywords:** MARCH-related protein, herpesvirus, caspase, Kaposi sarcoma

## Abstract

Viral infections lead to caspase activation as a cellular defense response. Some viruses overcome this response by encoding viral proteins that undergo caspase cleavage and, by various mechanisms, aid in cell survival. Kaposi sarcoma-associated herpesvirus (KSHV) latency-associated nuclear antigen acts as a pseudo-substrate for caspases-1 and -3, thereby interfering with the inflammatory and apoptotic response. Here, we screened the KSHV proteome for additional caspase cleavage sites. Using SitePrediction, many KSHV proteins with potential caspase cleavage sites were identified. Among the highest-scoring proteins was the early lytic protein, K5. Treatment of BJAB-K5-FLAG-expressing cells with ⍺Fas led to caspase processing of K5-FLAG. Using mass spectrometry, we determined that caspase cleavage of K5 occurs at D222. K5 was also cleaved by caspases when KSHV was induced to lytic replication. Interestingly, the expression of K5-FLAG significantly inhibited ⍺Fas-induced caspase-mediated cell death. To determine if K5 plays a protective role in KSHV-infected cells, iSLK cells infected with wild-type or K5-knockout BAC16 virus were induced to lytic replication. Although lytic induction showed no significant effect on the viability of wild-type-infected cells, the viability of K5-knockout cells decreased by 25%. Thus, K5 may protect KSHV-infected cells from caspase-mediated cell death during lytic replication. Interestingly, cleavage of K5 by caspases did not affect its previously reported ability to downregulate immune surface markers. These data suggest that K5 not only downregulates immune surface markers to help avoid immune recognition but plays an additional role in mitigating caspase-mediated cell death during lytic replication.

**IMPORTANCE:**

Kaposi sarcoma-associated herpesvirus (KSHV) is the etiological agent for Kaposi sarcoma, primary effusion lymphoma, multicentric Castleman’s disease, and KSHV inflammatory cytokine syndrome. Understanding how KSHV thwarts host defense responses is necessary to help develop strategies to treat these rare, yet deadly, diseases. We profiled potential caspase cleavage sites in the viral proteome *in silico* and found many viral proteins with high-scoring caspase cleavage sites. We follow up on this by demonstrating that K5 is, in fact, a substrate for caspases *in vitro* and *in cellulo* and provide data to suggest that K5 plays a role in obviating caspase-mediated cell death during lytic replication. The work described here furthers our understanding of the roles that KSHV proteins, like K5, play to prevent or divert the host apoptotic defense responses that involve host caspase activation that normally would lead to cell death.

## INTRODUCTION

Most viruses must overcome one of the most fundamental innate cellular defense responses: the induction of caspase-mediated cell death that leads to cell destruction before the virus can replicate. Viruses that infect eukaryotic cells have evolved means to prevent, inhibit, or redirect caspase activation upon viral infection. An early example of this was p35 protein from baculovirus which irreversibly inhibits caspases (1, 2) (for review, see reference 3). For chronic viruses like Kaposi sarcoma (KS)-associated herpesvirus (KSHV) or Epstein-Barr virus (EBV), mechanisms to thwart caspase activation are essential to allow the establishment of latency (3–5). KSHV, which is the cause of KS, primary effusion lymphoma (PEL), multicentric Castleman’s disease (MCD), and KSHV inflammatory cytokine syndrome, has evolved mechanisms to deal with multiple aspects of caspase activation and activity. For example, vFLIP protein of KSHV can prevent caspase-8 activation by preventing the death receptor complex from activating caspase-8 (6–8).

We previously showed that KSHV latency-associated nuclear antigen (LANA) encodes two caspase cleavage sites, one in the N-terminus and one in the C-terminus (9). These sites may act as decoy substrates for caspase-3 and caspase-1, aiding in the prevention of apoptosis and activation of the inflammasome, respectively. Also, the ORF63 lytic protein from KSHV acts as a viral homolog to NLRP1 and blocks NLRP1-mediated immune responses including caspase-1 activation and IL-1β and IL-18 production (10).

While inhibition of caspases by KSHV generally serves to allow lytic replication by preventing cell death, there are complexities to these interactions. For example, the increase in KSHV-induced caspase activity may benefit KSHV infection by antagonizing type I interferon responses (11, 12). Also, caspase-7 cleavage of the early lytic protein ORF57 (mRNA transcript accumulation [MTA]) appears to attenuate the level of virus production; this allows for optimal conditions during viral release that could aid in immune avoidance and increase virus dissemination (13). However, in general, it’s important for viral infection to limit caspase activity.

To follow up on previous studies of the caspase cleavage of LANA and ORF57 by caspases, we investigated the degree to which other KSHV-encoded proteins might be cleaved by caspases and the effects resulting from these interactions. We used SitePrediction (SP) to predict potential caspase cleavage sites in KSHV-encoded proteins. To our surprise, SP predicted that many KSHV proteins contain likely caspase cleavage sites. One particularly high-scoring site was identified in the early lytic protein, K5. K5 has previously been reported to downregulate several immune surface markers which aids in immune avoidance (14–17). We explored whether K5 is cleaved by caspases in KSHV-infected cells, and if so, what role this cleavage might play in KSHV pathogenesis and the regulation of immune surface marker expression. K5 was cleaved by caspases-3 and -8 in infected cells, and mechanistic studies suggest it can blunt cell death mediated by caspase-8 activation without losing its ability to downregulate immune surface markers.

## RESULTS

### Identification of predicted caspase cleavage sites in KSHV-encoded proteins using SitePrediction

We used SitePrediction (https://www.irc.ugent.be/prx/bioit2-public/SitePrediction/reference.php) (18) to screen 87 KSHV-encoded proteins for potential caspase cleavage sites and the class of caspases predicted to cleave these sites. Sixty-six KSHV proteins had SitePrediction scores, ranging from a low of 25 to a high of 2,787 (Table S1), predicting that they might be cleaved by caspases to varying degrees (the higher the score, the more likely it might be cleaved by caspases). Twenty-one KSHV proteins (K1, K4, K4.1, K4.2, K6, K7, K9, K12a, ORF11, ORF16/vBcl2, ORF18, ORF29a, ORF36, ORF41, ORF47, ORF52, ORF53, ORF58, ORF65, ORF66, and ORF69) were negative for predicted caspase cleavage sites (Table S1). Thirty-five KSHV proteins had caspase cleavage sites with a score over 100, and 13 of these had scores of 500 or higher, indicating a high likelihood of cleavage (Table 1). Table 1 lists, from highest to lowest, the top 35 scoring predicted cleavage sites found within the KSHV proteins that received a score over 100. Eighteen of these proteins contained multiple predicted sites with a score over 100, all of which are indicated in Table 1. Among these 35 proteins with sites scoring over 100 were ORF57/MTA and ORF73/LANA, two viral proteins that have previously been reported to be cleaved by caspases *in vitro* and in KSHV-infected cells (9, 13). This provides some confidence in the use of SitePrediction to identify additional caspase cleavage sites in KSHV proteins. That said, while we had previously confirmed two caspase cleavage sites in LANA, one close to the N-terminus DSVD^53^*GREC and the other near the C-terminus, MEVD^906^*YPVV (9), only the N-terminus cleavage site had a score >100 (score 687); the C-terminal site had a SitePrediction score of 28 for caspase-3. Thus, while these programs can be a useful guide for prioritizing additional KSHV proteins with caspase cleavage sites, some sites could be missed, and not all predicted sites will end up being bona fide sites. The known caspase cleavage site in ORF57 had the 4th highest SP score (1,742), while the N-terminal site in LANA had the 11th highest SitePrediction score (687). The top-scoring site predicted to be cleaved by caspases was within the ORF45 tegument protein with a score of 2,787 for caspase processing predicted at D99 (Table 1). Sites within two other KSHV proteins K10.5/vIRF-3 and K5/MIR2 had the second and third highest SitePrediction scores, respectively. Interestingly, 32 of the 35 proteins with scores over 100 are predicted by SitePrediction to be cleaved by executioner caspases (caspases-3, -6, and -7), although sites for initiator (caspase-8) and inflammatory (caspase-1) are also represented (Table 1). Also, certain sites are predicted to be cleaved by multiple caspases. For example, the N-terminal cleavage site in LANA is predicted to be cleaved by both initiator and executioner caspases. The high-scoring site in K5 is predicted to be cleaved by all classes of caspases (Table 1).

**TABLE 1:** KSHV protein sequences translated from the KSHV DNA sequence from the BCBL-1 PEL cell line (accession number U93872.2) were analyzed for caspase cleavage sites for caspases-1, -3, -6, -7, and -8 using SP (https://www.irc.ugent.be/prx/bioit2-public/SitePrediction/reference.ph)^*a*^^,*b*^

KSHV protein	Top scoring site by site prediction	SP score for top site and caspase class (I, Ex, Inf)	Other SP sites scoring over 100
ORF45*	DEED^99^*EDEE	2787 (Ex)	D101, D106, D112, D248, D277
K10.5/vIRF-3	EEVD^88^*DGAG	2679 (Ex)	None
K5/MIR2*	DEPD^222^*GGPN	1852 (I, Ex, Inf)	D37
ORF57/MTA*	**DETD** ^**33**^ *APTL	**1742 (I, Ex, Inf)**	D307
ORF38	VDVD^18^*GEPL	1649 (Ex)	None
ORF27*	VETD^97^*AI	1146 (Ex)	D291
ORF22*	DESD^648^*GLQS	985 (I, EX, Inf)	D336
ORF25*	VEGD^345^*KA	926 (Ex)	D280
ORF10	VEGD^208^*PE	863 (Ex)	None
ORF17*	VEKD^518^*APTP	728 (Ex)	D366
ORF73 LANA*	**DSVD** ^**53**^ *GREC	**687 (I, Ex)**	D324**^b^
ORF63*	TEYD^529^*ED	518 (Ex)	D5, D688
ORF62	VDLD^208^*ES	504 (Ex)	None
ORF70	VDAD^174^*AD	487 (Ex)	None
ORF59	ESPD^332^*SPPL	464 (Inf)	None
ORF67	VESD^116^*VY	424 (Ex)	None
ORF37*	DTLD^21^*GLTV	413 (Ex)	D341
ORF64*	DESD^1966^*TASG	392 (Ex)	D430,D998
K8*	IEED^28^*LS	320 (I, Ex)	D42
ORF54	GETD^93^*KD	297 (Ex)	None
ORF50/RTA	DSPD^408^*NPSS	276 (Inf)	None
K11/vIRF-2*	LAPD^27^*SPRP	274 (Inf)	D254
ORF6*	TEED^1039^*VI	262 (Ex)	D64, D400, D644
ORF21/TK*	TDDD^116^*SG	253 (Ex)	D45, D400
ORF75*	IEDD^184^*VI	252 (Ex)	D13
ORF34	EAVD^139^*GLCD	226 (Ex)	None
ORF4	SLTD^495^*SA	217 (I, Ex, Inf)	None
ORF71/vFLIP	TDVD*ALMS	214 (Ex)	None
K3/MIR1*	EDED^5^*VP	183 (Ex)	D232
ORF19	LELD^71^*RL	153 (Ex)	None
ORF20	EVLD^30^*SSSE	135 (Ex)	None
K14*	TDSD^178^*GLTV	128 (Ex)	D266
ORF74/GPCR	LDDD^14^*ES	125 (Ex)	None
ORF2	SSLD^94^*AALG	115 (Ex)	None
ORF49	QELD^31^*TL	101 (Ex)	None

^*a*^ The top-scoring site scoring over 100 for each protein is shown in column 2. Column 3 shows the score and the class of caspases (initiator caspase-8 [In], inflammatory caspase-1 [Inf], executioner caspases-3, -6, -7 [Ex]) predicted to cut that site. An asterisk (*) indicates that this protein has one or more other sites scoring over 100 and the cut sites are shown in column 4. Sites shown in bold have been experimentally verified.

^*b*^ Data for the acidic repeat region from 330 to 430 of LANA were excluded from the analysis due to the large number of DEED*DEED sites that score extremely high as caspase sites in SP. While these sites in LANA’s repeat region may be bona fide caspase sites, we did not find evidence for that in our previous study of LANA (9; #1520).

### K5-FLAG undergoes caspase cleavage in αFas-treated BJAB-K5F cells

**Fig 1:**
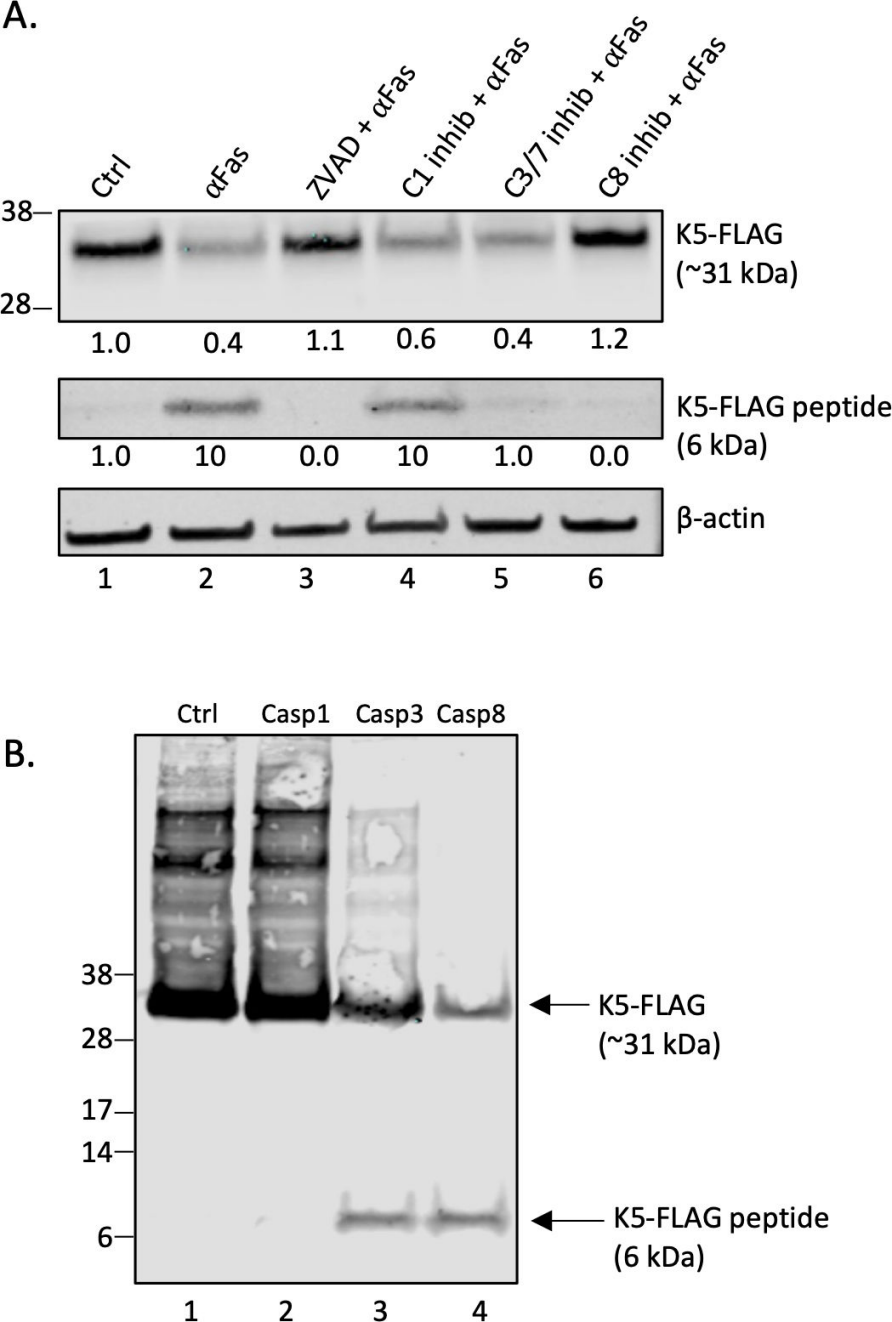
K5-FLAG undergoes caspase cleavage in αFas-treated BJAB-K5F cells and cell extracts. (**A**) Protein extracts were prepared from BJAB-K5F cells (300,000 cells/mL) and analyzed by western blot following a 24 h treatment with vehicle (DMSO) or different caspase inhibitors for 2 h prior to 10 ng/mL ⍺Fas treatment. Conditions studied were DMSO (lane 1), 10 ng/mL ⍺Fas (lane 2), 25 µM ZVAD with ⍺Fas (lane 3), 50 µM caspase-1 inhibitor with ⍺Fas (lane 4), 100 µM caspase-3/-7 inhibitor with ⍺Fas (lane 5), and 25 µM caspase-8 inhibitor with ⍺Fas (lane 6). The blots were probed with anti-FLAG and β-actin antibodies. The signal intensity of the intact K5-FLAG band in lane 1 was normalized with respect to β-actin, and the β-actin-normalized fold changes were calculated for the other treatments. A representative from two experiments is shown. (**B**) Cytoplasmic protein extracts from BJAB-K5F cells were prepared (without protease inhibitors), and then 8 µg of total protein in the presence of 50 mM dithiothreitol (DTT) was either untreated or treated with two units of caspase-1 (lane 2), caspase-3 (lane 3), or caspase-8 (lane 4) for 1.5 h and then stopped with SDS sample buffer followed by analysis by western blot. The blot was probed with anti-FLAG antibody. The intact K5-FLAG and K5-FLAG peptide fragments are indicated. The experiment was performed twice with similar results.

Because of its role in thwarting the immune response against KSHV, we were particularly interested in investigating the potential cleavage of K5/MIR2, an early lytic protein with the third highest SP score (DEPD^222^*GGPN) (Table 1). A primary function of K5 is to downregulate immune surface markers in KSHV-infected cells (14–17). To determine if the K5 protein might be susceptible to caspase cleavage in cells, we developed a K5-FLAG-expressing BJAB cell line (BJAB-K5F) with the 3×FLAG-tag at the C-terminus of the protein (Fig. S1). BJAB-K5F cells were treated with 10 ng/mL of αFas (a known apoptotic agent that works by activating initiator caspase-8 through the FADD pathway) in the absence or presence of a pan-caspase inhibitor (Z-VAD-FMK, called henceforth ZVAD) or caspase inhibitors directed at the three specific classes of caspases: caspase-1, caspase-3/caspase-7, and caspase-8. BJAB-K5F protein extracts were prepared and analyzed by immunoblot and probed using an anti-FLAG antibody (Fig. 1A). In untreated BJAB-K5F cells, K5-FLAG was detected near the expected relative molecular weight (MW) of 32 kDa (expected MW is 31 kDa based on 28 kDa for K5 plus approximately 3 kDa for 3×FLAG C-terminal tail) (Fig. 1A, lane 1). When cells were treated with αFas for 24 h, the levels of K5-FLAG protein decreased substantially (Fig. 1A, lane 2), and a new band was detected with the FLAG antibody at a relative molecular weight of about 6 kDa, indicative of caspase cleavage near the C-terminus of K5-FLAG (Fig. 1A, lane 2). In the presence of 25 µM ZVAD, the K5-FLAG levels were close to that of the untreated control and the 6 kDa band was no longer detected (Fig. 1A, lane 3), indicating that caspase cleavage of K5 had been blocked. A caspase-3/-7 inhibitor decreased the production of the 6 kDa fragment, and a caspase-8 inhibitor completely blocked cleavage of K5 (Fig. 1A, lanes 5 and 6). By contrast, a caspase-1 inhibitor did not block αFas-mediated production of the 6 kDa fragment (Fig. 1A, lane 4). Interestingly, while ZVAD and the caspase-8 inhibitor prevented the ⍺Fas-induced decrease in K5-FLAG and eliminated the production of the 6 kDa band, the caspase-3/-7 inhibitor did prevent the decrease in K5-FLAG, even though there were much lower levels of the 6 kDa band as compared to the αFas control (Fig. 1A, lane 5). This may indicate that cleavage is still occurring in the presence of the caspase-3/-7 inhibitor, but that subsequent degradation of the 6 kDa band also occurs under these circumstances.

To further investigate which class of caspases may be involved in the cleavage of K5-FLAG (initiator, executioner, or inflammatory), we made cytoplasmic extracts of K5-FLAG in the absence of protease inhibitors and then tested the different classes of recombinant caspases for cleavage of K5-FLAG as assessed by western blot (Fig. 1B). K5-FLAG did not undergo cleavage by the inflammatory caspase-1. However, K5-FLAG was clearly processed by the executioner caspase-3 and the initiator caspase-8, and both generated a 6 kDa FLAG band like that seen in αFas-treated BJAB-K5F cells. These results suggest that both executioner (caspase-3) and initiator (caspase-8) caspases can cleave K5-FLAG protein at the C-terminus. Interestingly, the highest SP scoring caspase site in K5 identified was at D222. Cleavage of K5-FLAG at D222 by caspases would generate an approximately 6 kDa K5-FLAG protein band like that observed by western blot (the calculated average mass for the C-terminal peptide with 3×FLAG is 6,311) (Fig. S1). Also, both caspase-3 and caspase-8 scored high in SP for cleavage at D222 (scores of 1,852 and 1,690, respectively).

### K5-FLAG is cleaved at D222 by caspase-8

**Fig 2:**
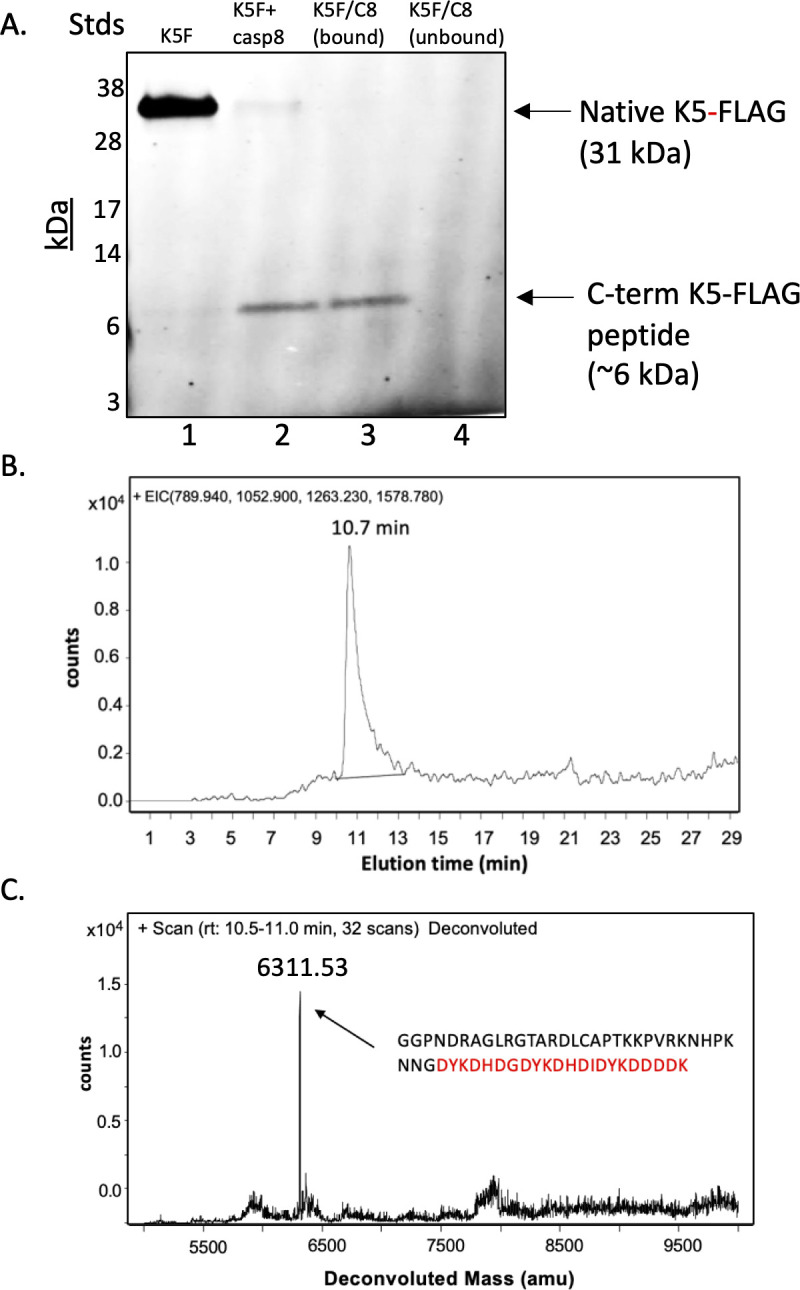
Purification of the K5-3×FLAG peptide and mass identification using RP/HPLC MALDI-TOF analysis. Protein extracts of BJAB-K5F cells were prepared using mPer, and K5-FLAG peptide was then purified with anti-FLAG beads. (**A**) Western blot for crude K5-FLAG protein (lane 1), caspase-8 treated K5-FLAG mPer extract (lane 2), bound material eluted from anti-FLAG beads using 8 M urea (lane 3), and the unbound flow thru fraction from anti-FLAG beads (lane 4). (**B**) Analysis of the purified K5-FLAG peptide by RP-HPLC/MS chromatography. The major ions predicted for the flag peptide produced following cleavage at D222 (789.8, 902.5, 1,052.8, 1,263.1, and 1,578.7) were extracted from the total ion chromatogram (TIC) and a peak containing all the ions was identified eluting at 10.7 min. Major ions for the other predicted peptides produced if cut after aspartic acid were not found. (**C**) Deconvolution of the peak identified in (**B**) revealed a molecular weight of 6,311.53. Predicted molecular weight of 6,310.70 for the peptide cleaved after D222 (amino acids 223–258).

To identify the site of caspase cleavage in K5-FLAG, we prepared protein extracts from BJAB-K5F cells (Fig. 2A, lane 1). Since caspase-8 was the most effective at cleaving K5 in extracts, we treated with caspase-8 for 2 h to allow processing of K5-FLAG, which revealed mostly processed K5-FLAG and a small amount of unprocessed K5-FLAG (Fig. 2A, lane 2). Following this, the cleaved FLAG peptide was purified using anti-FLAG beads. Western blot for FLAG showed that the peptide material eluted from the beads contained only the 6 kDa FLAG-containing peptide (Fig. 2A, lane 3). There was no peptide detected in the unbound fraction (Fig. 2A, lane 4). The intensity of the 6 kDa band generated was always substantially less than expected based on the intensity loss of the intact K5-FLAG after caspase-8 treatment. The reason for this is unclear but may be due to a relative inability of the anti-FLAG antibody to pick up the small peptide compared to that for larger proteins. However, it is also possible that subsequent degradation of the peptide occurs, leading to a loss of detectable peptide.

The sample containing the FLAG-peptide was dried by SpeedVac and then brought up in reverse phase high performance liquid chromatography (RP-HPLC) running buffer. The sample was then run on a C18 RP-HPLC column, and any bound peptides and proteins were eluted and run directly into an Agilent time-of-flight mass spectroscopy (MS/TOF) mass spectrometer to identify masses for any eluting peptides. The TIC scan was interrogated for peptide ions consistent with cuts at aspartate residues in the C-terminus of K5-FLAG (Fig. S2). Only peptide ions consistent with a peptide generated from cleavage at D222 (789.8^8+^, 902.5^7+^, 1,052.8^6+^, 1,263.1^5+^, and 1,578.7^4+^) were found (Fig. 2B) and deconvolution of the extracted ion peak gave a mass of 6,311.51 (Fig. 2C), which is in very good agreement with the predicted average mass of 6,310.70 for the D223-258-3×FLAG peptide. These data indicate that K5-FLAG is cleaved by caspase-8 at the highest scoring SP site (D222).

### Detection of the N-terminal cleavage fragment of caspase-cleaved K5-FLAG

**Fig 3:**
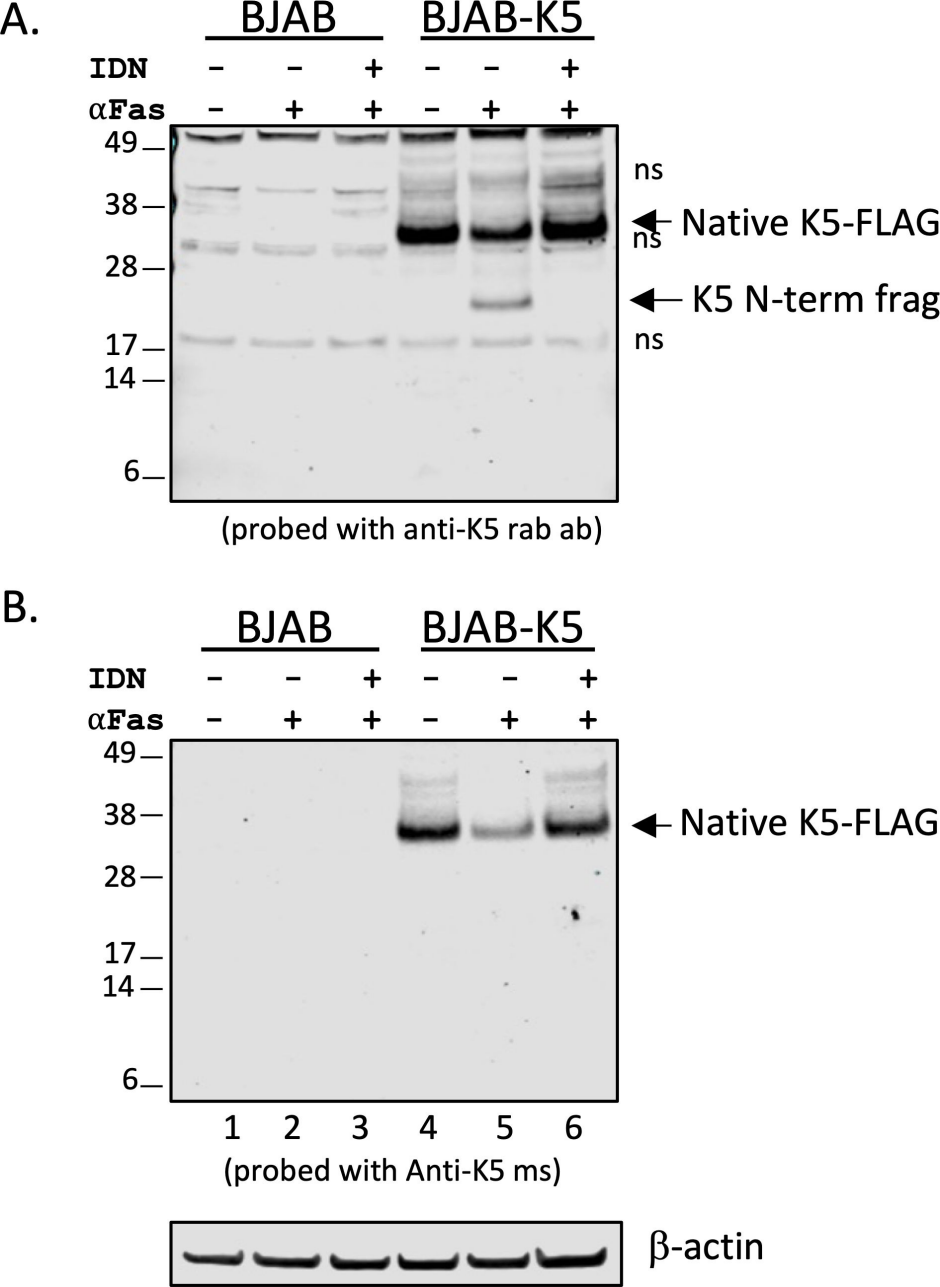
Analysis of BJAB and BJAB-K5F extracts by immunoblot using a K5 mouse or K5 rabbit antibody. BJAB or BJAB-K5F cells were treated with vehicle control, ⍺Fas (10 ng/mL), or IDN pan-caspase inhibitor (10 µM) for 2 h followed by treatment with vehicle of ⍺Fas. Protein extracts were prepared 18 h later, and samples were analyzed by immunoblot. (**A**) Blot probed with rabbit-anti-K5 antibody (anti-K5 rab ab) and (**B**) blot probed with mouse anti-K5 antibody (anti-K5 ms). Lanes 1–3 are BJAB lysates, and lanes 4–6 are BJAB-K5F lysates. ns indicates nonspecific bands. A representative of two similar experiments is shown.

We wanted to further confirm the cleavage of K5 through detection of the large N-terminal fragment that would be predicted to be generated following caspase cleavage at the C-terminus. To this end, we utilized a rabbit antibody custom prepared by GenScript that targets amino acids 204–217 of K5, which is in the C-terminal region of K5 but prior to the D222 caspase cleavage site. We first assessed whether this antibody was able to detect both K5 (as K5-FLAG) and the larger N-terminal fragment (amino acids 1–222) generated by caspase cleavage at D222 using extracts from BJAB and BJAB-K5F-expressing cells. Several nonspecific bands were detected in BJAB and BJAB-K5F protein extracts using this rabbit antibody (Fig. 3A, lanes 1–3). However, the rabbit antibody clearly detected K5-FLAG in the BJAB-K5F protein extracts (Fig. 3A, lanes 4–6). Treatment with αFas led to a decrease in the total K5-FLAG detected and revealed a new band around 25 kDa, indicative of the expected larger fragment after caspase cleavage (Fig. 3A, lane 5). When BJAB-K5F cells were first pretreated with the pan-caspase inhibitor emricasan (IDN-6556) (IDN) before addition of αFas, the 25 kDa band was no longer detected, and the levels of K5-FLAG were restored (Fig. 3A, lanes 5 and 6). The same blot was probed for K5-FLAG using an anti-K5 mouse monoclonal antibody (anti-K5 ms). This anti-K5 mouse antibody was able to detect K5-FLAG in the BJAB-K5F cells but not in BJAB cells (Fig. 3B, lanes 1 and 4). This antibody, however, did not pick up the 25 kDa band or the 6 kDa FLAG-containing band upon treatment with αFas, even though it could detect a decrease in the amount of full-length K5-FLAG, suggesting that it is only good at detecting intact K5-FLAG (Fig. 3B, lane 5). When cells were treated with the pan-caspase inhibitor IDN before addition of αFas, the loss of K5-FLAG, as determined using the mouse antibody, was prevented, again indicating caspase processing of K5 occurred following αFas treatment (Fig. 3B, lane 6). Together, these data indicate that K5-FLAG undergoes caspase cleavage near the C-terminus, resulting in a 25 kDa truncated K5 and a 6 kDa C-terminal FLAG fragment.

### KSHV K5 undergoes caspase cleavage in lytic PEL cells

**Fig 4:**
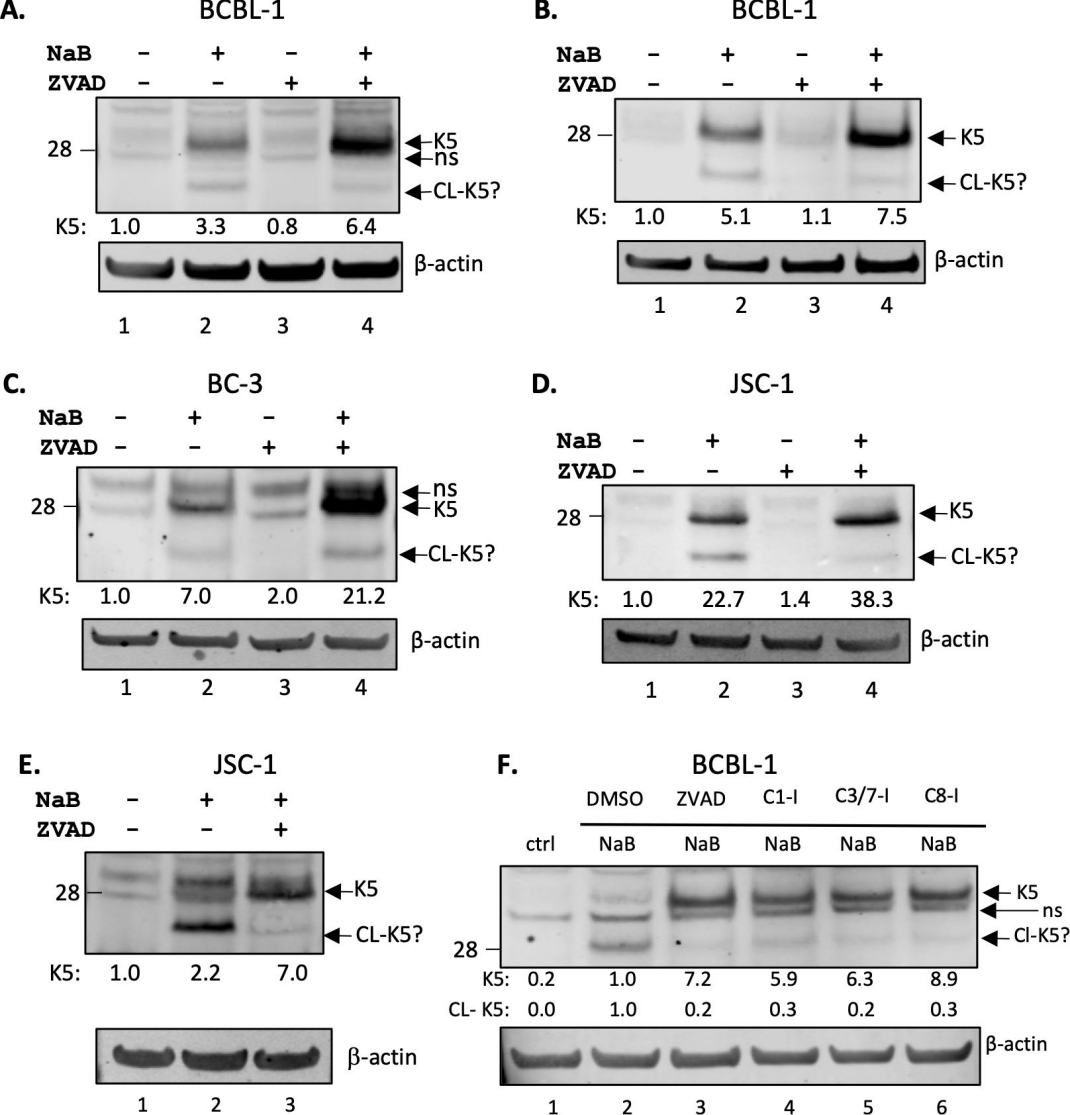
Caspase inhibition increases K5 protein levels in lytic PEL cells**.** (**A**) Cytoplasmic protein extracts and (**B**) nuclear protein extracts from BCBL-1 cells analyzed by western blot using rabbit K5 and β-actin antibodies. The protein lysates were extracted from BCBL-1 cells after 48 h of treatment with DMSO control and PBS control (lane 1), DMSO and 1 mM NaB (lane 2), 25 µM ZVAD and PBS (lane 3), or ZVAD and NaB (lane 4). Nonspecific bands detected near K5 are indicated as ns. (**C**) Cytoplasmic protein extracts from BC-3 cells analyzed by western blot using K5 and β-actin antibodies. Protein lysates were extracted from BC-3 cells after 48 h of treatment with DMSO control and PBS control (lane 1), DMSO and 1 mM butyrate (lane 2), 25 µM ZVAD and PBS (lane 3), or ZVAD and butyrate (lane 4). The ns indicates a nonspecific band detected near K5. (**D**) Nuclear and (**E**) cytoplasmic protein extracts from JSC-1 cells were analyzed by western blot using rabbit K5 and β-actin antibodies. Signal intensity of each protein with respect to β-actin and normalized to lane 1 is displayed under each blot. (**F**) Cytoplasmic protein extracts from BCBL-1 cells analyzed by western blot using K5 rabbit antibody. Protein lysates were extracted from BCBL-1 cells after 48 h of treatment with DMSO control and PBS control (lane 1), DMSO and 1 mM NaB (lane 2), 25 µM ZVAD and NaB (lane 3), 50 µM caspase-1 inhibitor and NaB (lane 4), 100 µM caspase-3/-7 inhibitor and NaB (lane 5), or 25 µM caspase-8 inhibitor and NaB (lane 6). Signal intensities of each protein with respect to β-actin and normalized to lane 2 are displayed under each blot. A representative experiment is shown for at least two separate experiments for each condition except JSC-1 cytoplasmic, which was done once.

Previous studies have established that lytic induction of KSHV-infected cells leads to caspase activation over levels that are seen in latency (9, 11–13). We therefore wanted to determine whether K5 undergoes caspase cleavage in KSHV-infected cells induced to lytic replication. To determine if K5 processing also takes place in the context of KSHV-infected PEL cells, we prepared cytoplasmic and nuclear extracts of BCBL-1 cells 48 h after lytic induction with sodium butyrate (NaB) in the absence or presence of the pan-caspase inhibitor ZVAD, then analyzed for K5 with rabbit anti-K5 antibody. K5 levels increased 3.3-fold in cytoplasmic extracts upon induction with NaB. In addition, a band running below intact K5 was observed at around 25 kDa, which is likely cleaved K5 (Cl-K5?) (Fig. 4A, lanes 1 and 2). Treating BCBL-1 cells with ZVAD alone had no discernible effect on background K5 levels (Fig. 4A, lane 3). However, when BCBL-1 cells were pretreated with ZVAD followed by lytic induction with NaB, full-length K5 levels nearly doubled, while the lower 25 kDa band was less intense, indicating that a substantial portion of K5 produced during lytic activation undergoes caspase cleavage (Fig. 4A, lanes 2 and 4). We also examined the nuclear extracts of BCBL-1 cells (which consist of both nuclear and plasma membrane proteins) using the NE-Per Kit from Thermo Fisher, since K5 is known to be shuttled to the inner plasma membrane from the endoplasmic reticulum (ER) (15) and has been found in the ER fraction of cells (19). K5 was also detected in the membrane fraction of NaB-treated BCBL-1 cells and increased fivefold over background levels from uninduced cells (Fig. 4B). K5 further increased to 7.5-fold over control in the presence of ZVAD plus NaB, while the levels of the lower band decreased. This again is consistent with caspase cleavage of K5 in the membrane protein extracts. In BC-3 cells, K5 levels increased 7-fold with NaB induction, but in the presence of ZVAD, K5 increased more than 21-fold over control and three times that seen with NaB alone (Fig. 4C, lane 4). By contrast to BCBL-1 cells, the lower band seen in NaB-treated BC-3 cells increased in intensity in the presence of ZVAD, although the ratio of the lower band to the upper band was measurably decreased (Fig. 4C, lanes 2 and 4). We also examined nuclear and cytoplasmic extracts of JSC-1 cells, which are coinfected with EBV and KSHV (20). NaB strongly induced K5 (almost 23-fold) in JSC-1 nuclear extracts when compared to the uninduced control (Fig. 4D, lane 2), and in the presence of ZVAD, the K5 levels increased to 38-fold over the control without NaB (and 1.4-fold over NaB alone). In cytoplasmic extracts, NaB increased full-length K5 2.2-fold with much of the K5 seen as the cleaved form (Fig. 4E, lane 2). In the presence of ZVAD, the K5 levels increased to sevenfold over the control (and 3.2-fold over NaB alone). These data provide good evidence that K5 undergoes processing near the C-terminus by caspases in PEL cells during lytic replication.

Further experiments were done to explore which caspases may be involved in the cleavage of K5 during lytic replication by using inhibitors for the specific caspases. BCBL-1 cells were treated with NaB without and with specific caspase inhibitors representing each of the three classes of caspases: inflammatory (caspase-1), apoptotic initiator (caspase-8), and apoptotic executioner (caspases-3/-7). These cell extracts were analyzed by western blot and probed for K5 expression using rabbit anti-K5 antibody (Fig. 4F). In control BCBL-1 cells, there was low K5 expression, as seen previously. Following lytic induction with NaB, we detected some K5 expression at the expected relative molecular weight of 28 kDa, but more protein was detected at the 25 kDa band. Again, ZVAD increased full-length K5 and decreased the levels of the 25 kDa band. Comparison of the three different caspase inhibitors used at their recommended optimal concentrations demonstrated that inhibitors of caspases-3/-7 and caspase-8 were the most effective at blocking cleavage and increasing native K5 protein (Fig. 4F). As we saw with BJAB-K5F extracts (Fig. 1), the caspase-1 inhibitor also had some activity but was the least effective of the caspase inhibitors, as there was less native K5 and more of the 25 kDa fragment than with the other three inhibitors (Fig. 4F). Overall, these data with the different caspase inhibitors suggest there is some caspase cleavage at the C-terminal site by caspases from all three classes of caspases. However, the accumulation of native K5 and substantial reduction of the 25 kDa band by caspase-3/-7 and −8 inhibitors provide evidence that the apoptotic executioner caspases-3/-7 and initiator caspase-8 are likely to be primarily responsible for the cleavage of K5 in lytic PEL cells.

### BJAB cells expressing K5-FLAG are resistant to caspase-mediated αFas-induced cell death

**Fig 5:**
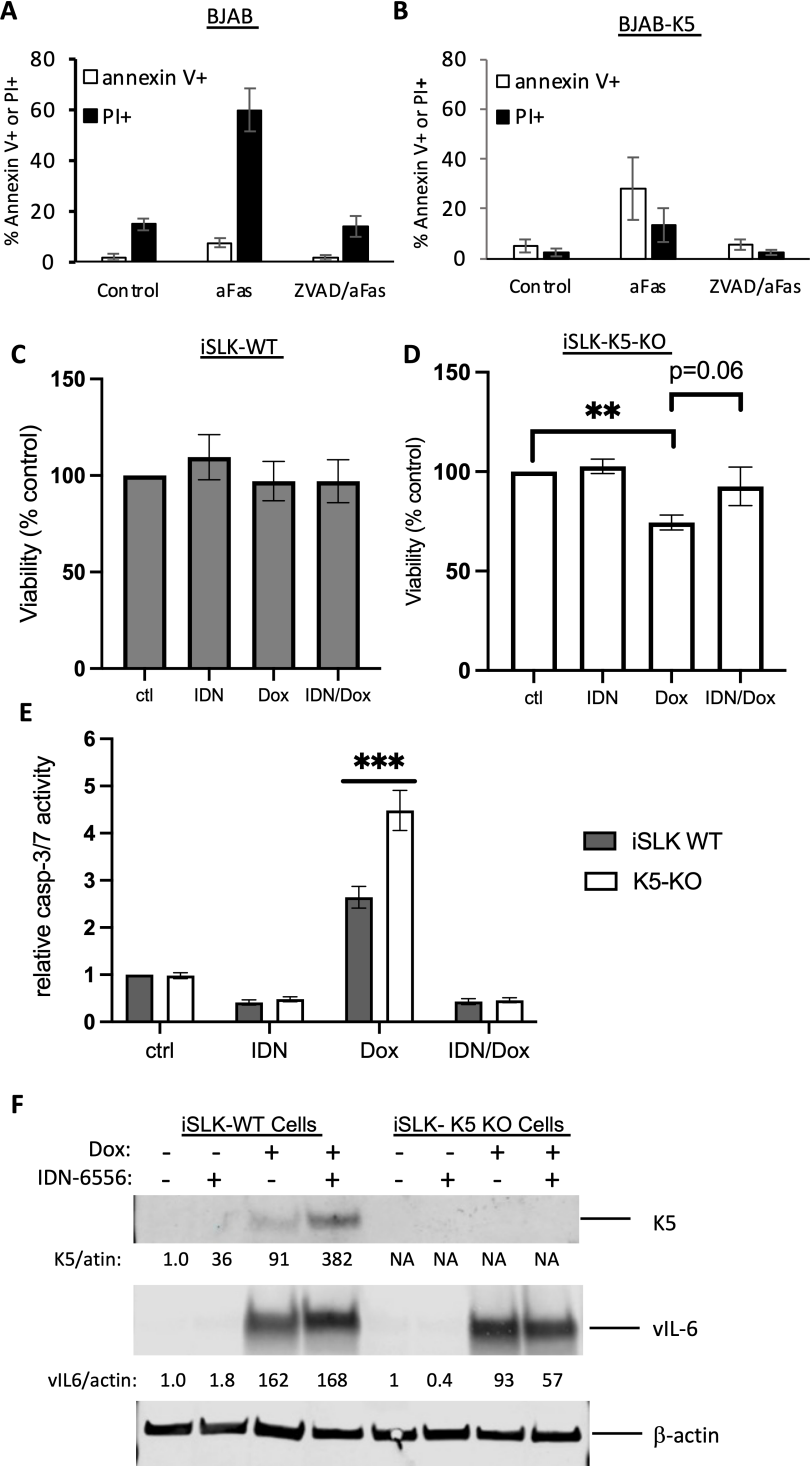
BJAB cells expressing K5-FLAG are resistant to caspase-mediated αFas-induced cell death, while K5 KO cells are susceptible to caspase-mediated cell death in cells induced to lytic replication by doxycycline (Dox). BJAB and BJAB-K5F cells were plated at 200,000 cells per milliliter and treated with PBS control, αFas (10 ng/mL), or αFas in the presence of 25 µM ZVAD. After 24 h, (**A**) BJAB cells or (**B**) BJAB-K5F were stained with annexin V (white bars) to assess live apoptotic cells and PI (black bars) to measure dead cells and analyzed by FACS. (**C**) iSLK-KSHV wild-type (iSLK-WT) and (**D**) K5-knockout (iSLK-K5-KO) cells were pretreated for 2 h with DMSO or IDN, then treated with diluted DMSO or 1 µg/mL Dox. After 48 h, floating cells were collected, and adherent cells were removed with trypsin and collected. The combined solutions of floating and adherent cells were counted using trypan blue staining, and cell viability was calculated. Shown are the averages and standard deviations for three separate experiments, **P < 0.01. (**E**) iSLK-KSHV WT (iSLK WT) and K5-knockout (K5-K0) cells were pretreated for 2 h with DMSO or IDN, then treated with 1 µg/mL Dox for 24 h. Lysates were made, and the activity of caspase-3/-7 was measured for WT lysates (dark bars) and K5-KO lysates (white bars). The results shown are the average of three separate experiments for each line, and the values are normalized to the activity of the WT control cells, ***P < 0.005. None of the other comparisons between iSLK-WT K5-KO cells are significant. (**F**) Western blot for K5 (using the mouse antibody) and vIL-6 (used as another lytic marker) in WT and K5-KO cells 48 h after treatment with Dox. The relative values for K5 and vIL-6 are indicated and are normalized to their respective controls.

The experiments with K5-FLAG indicated that K5 could be efficiently cleaved by caspase-3 and caspase-8. Caspase-8, an initiator caspase that activates downstream effector caspases such as caspase-3 and -7, can be activated by treatment of cells with αFas (21). We examined whether expression of K5-FLAG in BJAB cells may affect caspase-mediated cell death caused by αFas. To assess the activation of apoptosis and the level of cell death induced by αFas, annexin V and PI staining was done by fluorescence-activated cell sorting (FACS) analysis following treatment with αFas for 24 h to measure apoptotic live cells and dead cells, respectively. Treatment of BJAB cells for 24 h with αFas led to increased annexin V staining and substantial cell death (45% increase over control levels) (Fig. 5A), as compared to BJAB -K5 cells, which showed even more annexin V staining but much less cell death (10% over control levels) (Fig. 5B). This indicates that αFas triggers apoptosis in both lines (based on annexin V staining), but that the αFas-induced cell death is being prevented or delayed by K5-FLAG. The cell death induced by αFas was completely inhibited in the presence of ZVAD, demonstrating the death was caspase mediated.

### iSLK-KSHV K5-knockout cells are susceptible to caspase-mediated cell death in cells induced to lytic replication by doxycycline (Dox)

We were interested in knowing if K5, which is induced during lytic replication, might play a role in protecting KSHV-infected cells from caspase-mediated cell death. To address this question, we obtained wild-type (WT) and K5-knockout KSHV-infected iSLK cells (a kind gift from Dr. Jae Jung [22]). We first verified the level of expression of K5 transcripts in WT cells and compared that to the expression level in K5-knockout cells. The change in K5 gene expression was measured following induction of the cells with Dox, which leads to activation of an RTA-expressing plasmid in these cells. In WT cells, K5 transcripts on day 0 were about 10^4.2^ copies per microgram of RNA, and the levels steadily increased to over 10^7^ copies per microgram by 72 h (Fig. S3A). However, no transcripts were detected in the K5-knockout cell line, confirming the knockout line was not expressing K5 mRNA (Fig. S3A). In addition, we examined these cells for K5 protein expression, following induction with Dox, using the rabbit and mouse K5 antibodies. Using the rabbit antibody to K5, K5 was only detected in the WT-induced cells but not in the K5-knockout line, although the antibody picked up a strong nonspecific band just below K5 that was seen in all samples (Fig. S3B). We did not observe a truncated/caspase-cleaved form of K5 in the WT-infected cell extracts using the rabbit anti-K5 antibody, but this antibody reacted to a nonspecific band just below K5, possibly obscuring detection of a cleaved form (Fig. S3B). We also examined the extracts for the lytic ORF45 tegument protein. After Dox treatment, ORF45 was detected in WT and K5-knockout iSLK-KSHV cells at similar levels, suggesting similar lytic activation in both cell lines (Fig. S3D).

Previous studies have demonstrated that induction of lytic replication by Dox in iSLK-KSHV cells does not lead to cell death, even though caspase activation is observed (11). We wanted to determine if Dox (1 µg/mL) might induce cell death in cells lacking K5. To explore this, we treated the two lines with Dox without or with the pan-caspase inhibitor IDN (10 µM), which has been shown to inhibit caspase activity in the WT iSLK-KSHV lines (11). WT iSLK-KSHV-infected cells did not undergo significant cell death following lytic activation with Dox for 48 h (Fig. 5C). However, with the K5-knockout cells, approximately 25% of the cells died in this period as compared to the control cells (Fig. 5D). This Dox-induced cell death was mostly blocked in the presence of IDN, and the cell death in the IDN/Dox treatment was no longer significantly different from the uninduced control (Fig. 5D). These data suggest that K5 in iSLK-KSHV cells infected with WT KSHV can at least, in part, prevent caspase-mediated cell death during lytic replication. To determine if the level of caspase activity differed between the WT and K5-knockout cells following lytic induction with Dox, we measured the levels of caspase-3/-7 in lysates of these cells. In the uninduced cells, similar low basal activity was detected in both cell lines, and about half of the basal activity was blocked by IDN (Fig. 5E). However, Dox (1 µg/mL)-treated knockout cells showed greater caspase-3/-7 activity 24 h after treatment as compared to the Dox-treated WT-infected cells, and this activity was fully blocked by IDN (Fig. 5E). These data support the hypothesis that K5 may act as a substrate decoy, leading to less measurable activity in cell lysates. We also measured the levels of K5 in WT and K5-knockout cells following Dox treatment for 48 h. Using our mouse K5 antibody, K5 was detected in Dox-treated WT cells but not in K5-knockout cells (Fig. 5F). K5 levels in iSLK-KSHV WT cells increased more than threefold in the presence of IDN, implying caspase cleavage of K5 was occurring in Dox-treated cells. We also looked at the levels of vIL-6, another lytic protein. By contrast to K5, vIL-6 was detected in both cell lines, and the levels of vIL-6 did not substantially change in the presence of IDN in WT or K5-knockout cells (Fig. 5F). Together, these data indicate that the presence of K5 can delay or prevent caspase-mediated cell death in cells undergoing lytic replication, and that the absence of K5 can result in increased caspase-mediated cell death.

### K5 may reduce caspase-mediated cell death by delaying caspase cleavage of downstream caspase targets

**Fig 6:**
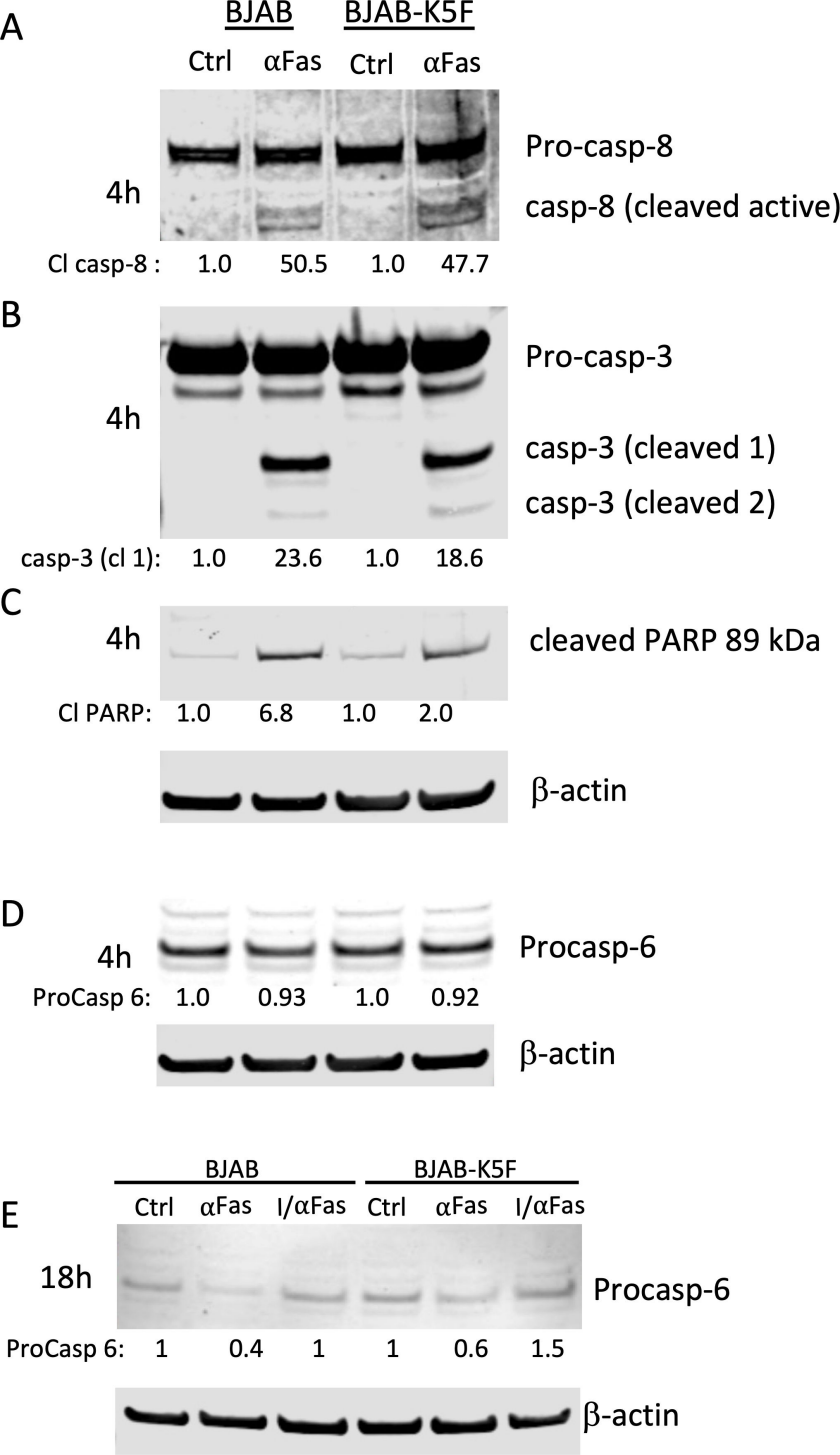
Downstream targets of caspase-3/-7 are cleaved less in BJAB-K5F cells. Radioimmunoprecipitation assay (RIPA) buffer protein extracts were prepared 4 h or 18 h after treatment of BJAB cells or BJAB-K5-FLAG cells with ⍺Fas (10 ng/mL), run on western blots, and analyzed for (**A**) caspase-8 and cleaved caspase-8, (**B**) caspase-3 and cleaved caspase-3, (**C**) the cleaved form of PARP (89 kDa), and (**D**) procaspase-6. Signal intensity of each protein of interest was normalized with respect to β-actin, and the value is displayed under each blot. Signal intensity of each protein with respect to β-actin and normalized to lane 1 is displayed under each blot. (**E**) An 18 h experiment was done with ⍺Fas treatment without or with IDN (10 µM) caspase inhibitor (labeled I/⍺Fas) and then analyzed for procaspase-6. A representative experiment of at least two is shown. The relative levels of procaspase-6 are shown after normalizing to β-actin.

Since we found that the presence of K5-FLAG decreased αFas-induced cell death, we measured the protein levels of caspase-8 and caspase-3 and the cleavage of their downstream targets such as PARP and caspase-6 in BJAB and BJAB-K5F cells. After 4 h of αFas stimulation, we detected similar caspase-8 activation based on western blot analysis which revealed the activation doublet 43/41 (Fig. 6A), suggesting K5-FLAG was not interfering with the initial activation of caspase-8 through the FADD receptor. Within the same samples, we also measured the activation of caspase-3 after 4 h, which occurs via caspase-8 cleavage of procaspase-3 during αFas treatment. Here, the induction of cleaved caspase-3 was somewhat lower (18.6-fold vs 23.6-fold) in the BJAB-K5F cells (Fig. 6B). We also examined the extent of PARP cleavage in αFas-treated BJAB and BJAB-K5F cells. PARP is cleaved by executioner caspases (3 and 7) and is a useful readout for caspase-mediated apoptosis (23, 24). The increase in cleaved PARP following αFas induction in BJAB K5-FLAG cells was less than one-third of that seen in BJAB cells (2.0 vs 6.8), suggesting that K5-FLAG may in fact interfere with downstream targets of caspases (Fig. 6C). We also assessed the activation of caspase-6, which occurs via caspase-3 cleavage of procaspase-6. We were not able to detect cleaved forms of caspase-6, so we analyzed the change in levels of procaspase-6 as an indirect measure. At 4 h post-αFas treatment, we detected similar decreases in procaspase-6 (Fig. 6D). We also looked at the levels of procaspase-6 after 18 h of treatment with ⍺Fas. At this time point, we did see a somewhat greater loss of procaspase-6 in BJAB cells as compared to the BJAB-K5F cells, suggesting greater processing in BJAB cells to active caspase-6 (Fig. 6E). The loss of procaspase-6 was prevented when αFas treatment was done in the presence of the pan-caspase inhibitor IDN, indicating a caspase-dependent conversion of procaspase-6 to its active forms (Fig. 6E). Taken together, these data suggest that K5-FLAG may delay caspase-3/-7-mediated events and thus delay caspase-dependent cell death.

### Truncated GFP-K5 still can downregulate the immune surface markers MHC-1, ICAM-1, and B7-2

**Fig 7:**
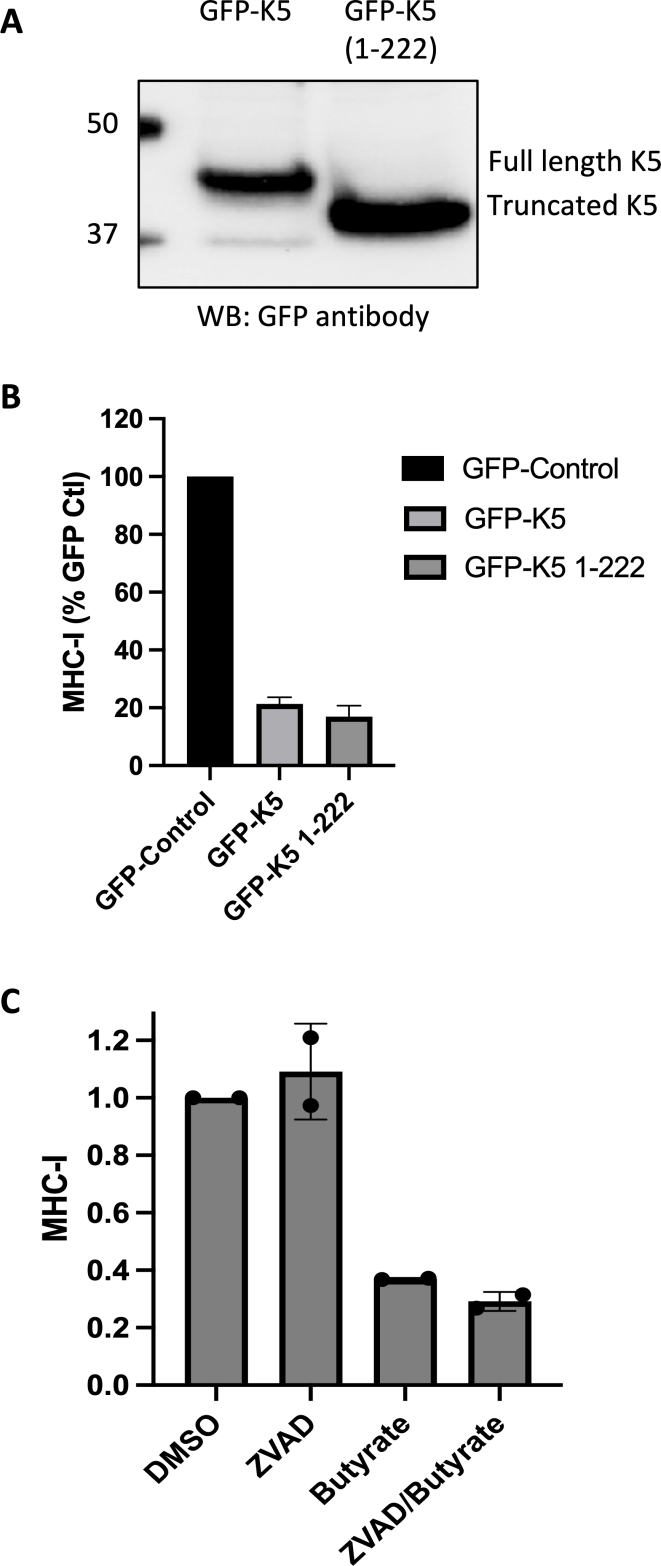
GFP-K5 and truncated GFP-K5 induce the downregulation of MHC-I. (**A**) Immunoblot for GFP-K5 and truncated GFP-K5 detected by using GFP antibody. (**B**) MHC-I surface expression in HEK-293T cells measured by FACS analysis 28 h after transfection with GFP-control vector, GFP-K5 plasmid, or truncated GFP-K5 (GFP-1-222) plasmid. (**C**) MHC-I surface expression in BCBL-I cells measured by FACS analysis following treatment for 48 h with DMSO control, ZVAD (25 µM), NaB (1 mM), or ZVAD then NaB. ZVAD was added 2 h prior to treatment with NaB. The data plotted in B are the average and standard deviation from three separate experiments, while in C, the data are the average of two experiments, with each value shown as a dot.

Based on our data, the caspase cleavage site identified in K5 yields a large N-terminal fragment of 25 kDa (predicted MW of 24.4 kDa) and a small peptide of about 3.6 kDa (or 6 kDa in the case of K5-FLAG). We decided to explore if loss of the C-terminal peptide due to caspase cleavage affects the ability of K5 to downregulate immune surface markers, which is considered a primary function of K5 (14–16, 22, 25). We developed plasmids expressing GFP-K5 or a truncated form of GFP-K5 representing the caspase-cleaved large fragment, designated as GFP-K5-D222, and then measured by FACS analysis their ability to downregulate major histocompatibility complex class I (MHC-I) in HEK-293 cells. GFP-K5 and GFP-K5 D222 were both well expressed (Fig. 7A). Also, both were able to significantly downregulate MHC-I (Fig. 7B). Although we explored the effect of GFP-K5s on the surface markers ICAM-1 and B7-2 in these cells, the levels of expression of these surface markers in control cells were too low to discern clear differences between the GFP-K5 activity. We also evaluated the effect of the GFP-K5s on MHC-I, ICAM-1, and B7-2 expression in BJAB cells, since these cells have higher expression of ICAM-1 and B7-2. In this case, as with HEK-293 cells, both GFP-K5 and GFP-K5-D222 downregulated MHC-I to similar extents. Moreover, they were equally effective at downregulating ICAM-1 and B7-2 in BJAB cells (Fig. S4). We also explored whether blocking caspase processing of K5, in the context of virus-infected cells, might affect the extent of downregulation of MHC-I in BCBL-1 cells during lytic replication. In two separate experiments, pretreatment of the cells with ZVAD had little effect on the downregulation of MHC-I in lytic BCBL-1 cells, going from an average of 63% downregulation by NaB alone to 71% downregulation in the presence of ZVAD and NaB (Fig. 7C). Overall, these data suggest that caspase cleavage of K5 does not substantially impair its ability to downregulate immune surface markers, and that the main advantage of this cleavage for KSHV may be to thwart caspase-mediated cell death during lytic replication.

## DISCUSSION

One of the main cellular defenses against viral pathogens is caspase-mediated cell death, where cells either undergo apoptosis, pyroptosis, or necroptosis upon infection (3). To survive infection, viruses must find ways to prevent these forms of caspase-mediated cell death to facilitate their replication and survival. Several comprehensive reviews have been published that describe the variety of ways in which human and non-human viruses evade caspase-mediated cell death (1, 3, 4, 26). The most direct way is by encoding viral proteins that directly inhibit caspases, as is the case with the baculovirus-encoded P35 (reviewed in reference 1). However, cleavage of viral proteins by caspases may have a variety of other effects, including production of cleavage products with novel activities. For some viruses, cleavage of viral proteins by caspases has been shown to lead to products that attenuate viral replication (13, 27, 28). Interestingly, for certain other viruses, some viruses (for example, Aleutian mink disease virus [AMDV], human papillomavirus [HPV], severe acute respiratory syndrome coronavirus-2 [SARS-CoV-2], etc.), caspase cleavage products have been shown to enhance viral replication, for example, by enhancing nuclear translocation of viral proteins (reviewed in reference 3).

Previous studies by our group and others showed that KSHV encodes at least two proteins, LANA and ORF57, that are cleaved by caspases. Cleavage of LANA was shown to inhibit caspase activity (9), while evidence was provided that cleavage of ORF57 could yield a cleavage product that reduces lytic replication (13). In this study, we used SitePrediction to identify additional KSHV-encoded proteins that may undergo caspase cleavage. Using SitePrediction, the top 10 KSHV proteins predicted to be susceptible to caspase cleavage were ORF45, K10.5, K5, ORF57, ORF38, ORF27, ORF22, ORF25, ORF10, and ORF17; these all scored higher than the already confirmed N-terminal caspase-3 cleavage site in LANA (D53). In this work, we confirmed that K5 is indeed cleaved by caspases in PEL cells undergoing lytic replication. K5 is an early lytic KSHV protein that, along with K3, downregulates immune surface markers during lytic replication (14–17, 25). Utilizing K5-FLAG protein expressed in lentivirus-infected BJAB cells, we showed that K5 is cleaved *in vitro* at D222 by caspase-8 and caspase-3 but not by caspase-1. While αFas treatment led to significant caspase-mediated processing of K5-FLAG, expression of K5-FLAG significantly decreased the extent of caspase-mediated cell death induced by αFas, an activator of caspase-8. In KSHV-infected iSLK cells exposed to Dox to induce lytic replication, knockout of K5 led to caspase-mediated cell death that does not occur in WT KSHV-infected cells under the same conditions (11). Our data suggest that K5 contributes to the prevention of cell death during lytic replication of KSHV.

We originally became interested in K5 when we found that the immune modulator, pomalidomide, could prevent the downregulation of immune surface markers caused by K5 (29). We initially postulated that following their activation by pomalidomide, caspases may cleave and inactivate K5, resulting in increases in immune surface markers. However, we found that even after truncation at the D222 caspase cleavage site, K5 was able to efficiently downregulate immune surface markers. Consistent with this, others have shown that deletion of the C-terminal tail (amino acids 233–256) of K5 does not significantly alter its ability to downregulate MHC-I, ICAM-1, or interferon (IFN)-gamma receptor 1 (25). We also found that blocking caspase activity in KSHV-infected cells undergoing lytic replication did not significantly change the downregulation of surface markers. These data suggest that caspase cleavage of K5 at the C-terminus does not significantly impair its ability to downregulate immune surface markers.

While caspase-mediated cell death is typically associated with apoptosis (extrinsic and intrinsic), it is now appreciated that caspases also play a role in other types of cell death including pyroptosis, necroptosis, and necrosis (30–36). Given that caspase cleavage of K5 does not reduce its ability to downregulate immune surface markers but reduces caspase activity, we hypothesize that KSHV has evolved to encode caspase cleavage sites as a mechanism to mute the overall caspase response, therefore effectively blocking the cellular antiviral strategy of caspase-mediated cell.

Interestingly, our analysis using SitePrediction revealed that the KSHV proteome includes many caspase cleavage sites that could be targets for different caspase classes, and extending the observations here, one can postulate that this is a general mechanism for KSHV to divert caspases away from their usual functions, which would otherwise thwart viral replication and persistence. Although we did not fully explore which types of caspase-mediated cell death were inhibited by K5, we did learn that it can prevent αFas-mediated cell death, which usually goes through the extrinsic pathway of apoptosis by activating caspase-8, followed by subsequent activation of extrinsic caspases (caspase-3, -6, -7) and cleavage of PARP (23, 24). The increase in the amount of cleaved PARP was greater in BJAB cells than in BJAB-K5-FLAG-expressing cells, although K5-expressing cells had higher baseline levels of cellular cleaved PARP to begin with. We also saw a delay in procaspase-6 processing in K5-FLAG-expressing cells. Caspase-6, which degrades lamin A during apoptosis, can be directly activated by caspase-3 (37), and in K5-FLAG-expressing cells, the extent of caspase-6 activation also appeared to be impaired. Our data suggest that K5 does not substantially impair caspase-8 or caspase-3 activation by αFas in cells but instead appears to decrease the cleavage of downstream cellular targets of caspase-3, like PARP and caspase-6, perhaps by acting as a decoy substrate. This is consistent with the finding that K5-knockout cells have higher caspase activity than the cells expressing K5. The evidence points to K5 acting as a decoy substrate for caspase-3, although other mechanisms may be in play and require further investigation.

Although we demonstrate a role for K5 in preventing caspase-mediated cell death, it remains possible that the cleaved forms of K5 or other KSHV proteins play other important roles in viral replication, possibly by conferring new functions to these proteins. Previous work by others has described a role for caspase activation in enhancing KSHV infection by preventing type 1 interferon responses (11, 12). It is possible that one or more of the other KSHV proteins susceptible to caspase cleavage may play a role in this response to caspase activation. Both KSHV-encoded K11/vIRF-2 and K10.5/vIRF-3 have high SitePrediction scores for caspase cleavage sites (see Table 1), and perhaps caspase cleavage of these proteins may play a role in altering the interferon response. Interestingly, K10.5/vIRF-3, which had the second highest SitePrediction score at D88 (score 2,679), is a latent protein that is only expressed in KSHV-infected B cells like those seen in PEL and MCD (38). K10.5/vIRF-3 has already been shown to antagonize apoptosis by inhibiting p53 and has been shown to decrease caspase-3 activation (39, 40). The presence of a caspase cleavage site in vIRF-3 and vIRF-2, if functional, could provide additional mechanisms by which these viral proteins may disrupt or redirect caspase-mediated events and is ripe for future studies.

In summary, in this study, we show that KSHV K5 can be cleaved by caspases and that by reducing caspase activity, this cleavage provides a mechanism by which KSHV thwarts cellular innate immunity. We also describe predicted caspase cleavage sites in many other KSHV proteins. Analysis of these interactions may yield further insights on the complex interplay between KSHV and host cells.

## MATERIALS AND METHODS

### Identifying potential caspase cleavage sites in KSHV proteins

SP (18) is a web-based program designed to predict caspase cleavage sites. SitePrediction predicts cleavage sites for caspases-1, -3, -6, -7, and -8, and it was utilized for this study. As of this writing (January 2025), SitePrediction is still available for public use at https://www.irc.ugent.be/prx/bioit2-public/SitePrediction/. We initially utilized different programs (Cascleave and Cascleave-2), but these are no longer freely accessible online. Eighty-seven different KSHV-encoded proteins were analyzed by SitePrediction, and the prediction scores were ranked from highest to lowest.

### Caspases and caspase inhibitors

Recombinant human caspase-1 (Cat #CC126), caspase-3 (Cat #CC119), and caspase-8 (Cat #CC123), as representatives of the three different classes of caspases, were from Sigma Aldrich-Calbiochem (St. Louis, MO). Caspase-1 inhibitor (Cat #400015), caspase-3/-7 inhibitor (Cat #218832), caspase-8 inhibitor (Cat #218759), cell-permeable pan-caspase inhibitor ZVAD-FMK (Cat #627610), and αFas (Cat #05-201) were from EMD-Millipore Sigma. The pan-caspase inhibitor IDN-6556 (Cat #S7775) was from Selleck Chemicals. All inhibitors were dissolved into 100% cell culture-grade dimethyl sulfoxide (DMSO) and stored at −20°C.

### Cells, cell culture, and reagents

BJAB**,** BC-3, and JSC-1 cells were from the American Type Culture Collection (Manassas, VA). BCBL-1 cells were obtained from the NIAID AIDS Research and Reagent Program (Rockville, MD). Cells were cultured in RPMI 1640 medium (Life Technologies, Carlsbad, CA) with 15% heat-inactivated fetal calf serum (HyClone, Waltham, MA) and 1% penicillin-streptomycin-glutamine (1,000 units/mL penicillin, 10,000 µg mL^−1^ streptomycin, 29.2 mg/mL L-glutamine) (Life Technologies). BJAB cells constitutively expressing K5-FLAG cells were prepared using lentivirus encoding a puromycin resistance marker as well as full-length K5 containing a 3×FLAG C-terminal tag (9,332 bp total) from Sigma. Lentivirus was electroporated into BJAB cells, and cells expressing K5-FLAG, driven by EF1A promoter, were selected with puromycin. Confirmation of K5-FLAG expression was done using an antibody to 3×FLAG (Cat #F1804) from Sigma as well as a mouse monoclonal antibody toward K5 (328C7), which was obtained from Drs. Koichi Yamanishi and Keiji Ueda (Osaka University Medical School, Japan) (19). WT and K5-knockout-infected iSLK-KSHV cells were obtained as a kind gift from Dr. Jae Jung’s group (22). Quantitative PCR was carried out as described previously, and the primers used for K5 and the controls are listed (Fig. S5). A rabbit K5 antibody directed toward a K5 peptide, amino acids 206–218, was generated by GenScript (Piscataway, NJ). A monoclonal β-actin antibody (Cat #A5441) and sodium butyrate, which was used to induce lytic replication in KSHV-infected cells, were from Sigma-Aldrich (St. Louis, MO). An antibody directed toward ORF45 was from Abcam (Cambridge, MA, Cat #ab36618). Antibodies to caspase-8 (Cat #9496, rab), caspase-7 (Cat #9492, rab), full-length caspase-6 (Cat #9762, rab), cleaved caspase-6 (Cat #9761, rab), caspase-3 (Cat #14220, rab), PARP (Cat #9532, rab), and cleaved PARP (Cat #9741, rab) were from Cell Signaling (Boston, MA). A monoclonal antibody to KSHV ORF2/vIL-6 was prepared as described previously (41). A synthetic peptide encompassing amino acids 223–278 of K5-FLAG was prepared by Vivitide (Gardner, MA).

### Purification of K5-FLAG peptide following caspase cleavage

K5-FLAG underwent caspase cleavage by caspases-3 and -8 but not caspase-1. To identify the site of caspase cleavage, the approximately 6 kDa FLAG-containing peptide generated following treatment with caspase-8 and detected by western blot was purified from anti-FLAG-labeled magnetic beads from Sigma (Cat #M8823). Protein extracts of K5-FLAG-expressing cells were prepared using mPer protein extraction reagent from Pierce and done in the absence of protease inhibitors. The mPer extracts were then treated with caspase-8, and the generated FLAG-peptide was captured using the anti-FLAG beads. The bound peptide was eluted with 8 M urea and then dialyzed against distilled water to remove the urea. The solution was then dried by SpeedVac and put up into RP-HPLC/MS running buffer (0.1% formic acid and 0.02% trifluoroacetic acid). The solution was injected onto a VyDac C18 column (Cat #218TP5205) and eluted with an acetonitrile (ACN) gradient (containing 0.1% formic acid and 0.02% trifluoroacetic acid). Analysis was done for 5 min at 0% ACN followed by a 2.5%/min increase for the next 30 min. Peptides were analyzed in-line with an Agilent MALDI-TOF mass spectrometer detector and were then identified by extracting the predicted molecular ions for peptides generated from the K5-FLAG C-terminus following caspase cleavage after the various aspartic acid residues (Fig. S2).

### Cell death and FACS analysis

To induce extrinsic apoptosis through the FADD pathway, we treated BJAB and BJAB-K5-FLAG cells (200,000 cells/mL) with 10 ng/mL of αFas antibody in the presence and absence of 25 µM ZVAD-FMK for 24 h. Cells were stained with annexin V and PI to assess live apoptotic cells and dead cells, respectively, by FACS analysis as described previously (42). Cell death that was prevented with ZVAD-FMK was considered caspase-mediated cell death.

### Immunoblotting

Nuclear and cytoplasmic extracts as well as mPER extracts were prepared as described previously (9) and were made in the presence of protease inhibitors (Halt Protease Inhibitors Cocktail Kit, Pierce) and 5 mM EDTA unless otherwise indicated. Protein concentrations were determined using the bicinchoninic acid (BCA) assay (Pierce), and samples were separated on 4-12% NuPAGE gels and transferred to nitrocellulose membranes by iBlot (Life Technologies). Membranes were probed with antibodies directed toward β-actin, 3×FLAG, K5, and ORF45 as indicated in the text. Blots were then incubated with appropriate secondary antibodies conjugated to alkaline phosphatase and visualized using stabilized Western Blue substrate (Promega) or with a goat anti-mouse or rabbit IR700 or IR800 secondary antibody (diluted 1:10,000) as indicated. Membranes exposed to LI-COR secondary antibodies were scanned using a LI-COR Odyssey infrared scanner. Images were analyzed using Image Studio 2.1 software.

### Transfection of HEK-293 cells

HEK-293 cells were plated at 12 × 10^5^ cells per well in a six-well plate. After 24 h, the cells were transfected using Genjet reagent (Cat #SL100488) from SignaGen (Frederick, MD) as per the manufacturer’s protocol. Briefly, DNA (Ctrl, WT-K5, or M-K5-expressing plasmids) and Genjet reagent were diluted separately by adding 2 µg DNA or 6 µL Genjet reagent to 50 µL high-glucose Dulbecco’s modified Eagle’s medium (DMEM) (Thermo Fisher, Cat #11965092). The DNA mix was then added to the Genjet mix to prepare the transfection mix and incubated at room temperature for 15 min. One hundred microliters of the transfection mix was then added dropwise to each well containing 1 mL of complete media. Transfection media was removed and replaced with complete media after 5 h. Surface markers were analyzed 28 h post-transfection as described previously (29).

### Transfection of BJAB cells

Transfections of BJAB cells with Ctrl, WT-K5, or M-K5-expressing plasmids were performed using 4D-Nucleofector X Unit (Lonza), with program SF at EN-150, as per the manufacturer’s protocol. Briefly, cells were washed with phosphate-buffered saline (PBS and resuspended at 5 × 10^6^ cells per 100 µL SF nucleofection buffer. A total of 1.25 µg of the DNA was then added to 100 µL nucleofection buffer and transferred to a cuvette for nucleofection. Nucleofected cells were incubated in the cuvettes for 10 min at room temperature, after which 500 µL prewarmed complete media were added, and the cells were then transferred to six-well plates. Cells were cultured for 28 h before performing surface marker analysis by flow cytometry, as described previously (29).

### Caspase activation assay

In a 96-well plate, iSLKK cells were plated at 10,000 cells per well and kept overnight. Then the cells were pretreated with DMSO or 10 µM IDN-6556 for 2 h, then treated with DMSO or 1 µg/mL doxycycline for 20 h. Caspase activity was measured using the Caspase-Glo 3/7 Assay System (Promega, G8091), according to the manufacturer’s protocol. Technical duplicates were averaged and normalized to cell viability and DMSO control.

### RNA extraction and RT-qPCR

Total RNA was extracted with Direct-zol RNA Miniprep Kit with on-column DNase I digestion (Zymo Research #R2053). Between 0.5 and 1 µg of total RNA was used for reverse transcription with ReverTra Ace qPCR RT Master Mix (Toyobo #FSQ-101), and quantitative PCR (qPCR) was performed with Thunderbird Next SYBR qPCR Mix (Toyobo #QPX-201) and StepOnePlus real-time PCR system (Thermo Fisher), following the manufacturer’s instructions. Any targets with a Ct of <34 were labeled as “not detected” or “nd.” Copy-level abundance was determined using standard curves generated using purified genomic stocks (KSHV bacterial artificial chromosome, BAC16). Absolute copy numbers of genomic stocks were determined using digital droplet PCR (Bio-Rad QX600).

### Statistical tests

When appropriate, statistical analysis was carried out to determine significance using a two-tailed Student’s *t*-test.

## Data Availability

All data for this study are contained in the article and the supplemental material.

## SUPPLEMENTAL MATERIAL

Supplemental figures jvi.00622-25-s0001.pdf Figures S1 to S5.

Table S1 jvi.00622-25-s0002.pdf KSHV protein sequences translated from the KSHV DNA sequence from BCBL-1 were analyzed for caspase cleavage sites for caspases 1, 3, 6, 7, and 8 using site prediction (SP).

ASM does not own the copyrights to Supplemental Material that may be linked to, or accessed through, an article. The authors have granted ASM a non-exclusive, world-wide license to publish the Supplemental Material files. Please contact the corresponding author directly for reuse.
