# Supplementary material for: Caspase cleavage of Kaposi sarcoma-associated herpesvirus proteins: a role for K5 in preventing caspase-mediated cell death during lytic replication
Source: J Virol. 2025 Aug 29;99(9):e00622-25. doi: 10.1128/jvi.00622-25 (PMC12456141; doi:10.1128/jvi.00622-25)

Supplemental Fig. S1

K5-FLAG:
MASKDVEEGVEGPICWICREEVGNEGIHPCACTGELDVVHPQCLSTWLTVSRNTACQMCRVIYRTRTQWRSRL
NLWPEMERQEIFELFLLMSVVVAGLVGVALCTWTLLVILTAPAGTFSPGAVLGFLCFFGFYQIFIVFAFGGICRVSG
TVRALYAANNTRVTVLPYRRPRRPTANEDNIELTVLVGPAGGTDEEPTDESSEGDVASGDKERDGSSGDEPD^{222}*****
GGPNDRAGLRGTARTDLCAPTKKPVRKNHPKNNGDYKDHDGDYKDHDIDYKDDDDK
(average molecular weight: 30,651)

K5-FLAG C-terminal peptide:
GGPNDRAGLRGTARTDLCAPTKKPVRKNHPKNNGDYKDHDGDYKDHDIDYKDDDDK
(average mol. Weight of 6311)

Fig. S1: (A) Amino acid sequence for K5-FLAG expressed in BJAB cells. The 3X Flag sequence is shown in red. The predicted caspase cleavage site found using SitePrediction is indicated with an asterisk at D222*. (B) sequence of the predicted K5-FLAG peptide following caspase-cleavage at D222.

Supplemental Fig. S2

Average mass ions	Peptide 239-278 4715.02	Peptide 228-278 5870.29	Peptide 223-278 6310.70	Peptide 220-278 6652.01	Peptide 215-278 7055.36	Peptide 211-278 7583.92	Peptide 206-278 8013.35	Peptide 200-278 8617.87
3+	1572.67	1957.76	-					
4+	1179.76	1468.57	1578.68	1664.00	1764.84	1896.98		
5+	944.00	1175.06	1263.14	1331.40	1412.07	1517.78	1603.67	1724.57
6+	786.83	979.38	1052.78	1109.67	1176.89	1264.99	1336.56	1437.31
7+	674.57	839.61	902.52	951.29	1008.91	1084.44	1145.76	1232.12
8+			789.84	832.50	882.92	948.99	1002.67	1078.23
9+						843.66	891.37	958.54
								862.78

Fig. S2: List of major ions expected for peptides generated by cleavage after aspartic acid.. These ions were used to interrogate the eluted peptide from anti-Flag beads. Only peptide ions for peptide cut at D222 (amino acids 223-278) were detected in the eluting peptides.

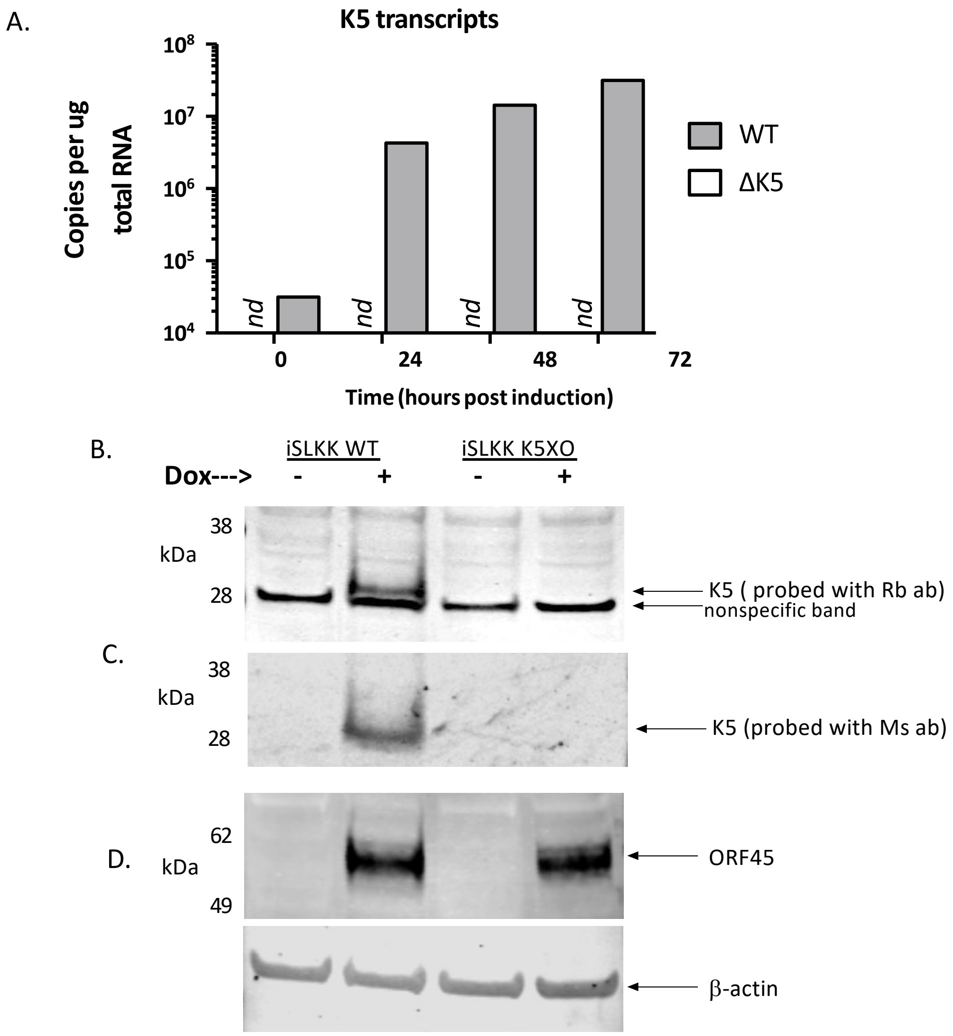

Fig. S3: (A) qPCR on transcripts from WT iSLKK and delta-K5 iSLKK using a standard curve with known genome and KSHV BAC16 genome concentrations to quantify transcripts present (nd = ct > 34, nothing detected based on melt curve). K5 transcripts are detected in uninduced WT cells and increase upon Dox induction. No transcripts were detected in the delta-K5 iSLKK cells. Western blot of cytoplasmic lysates from uninduced and induced (Dox) WT and delta-K5 iSLKK cells for detection of K5 using the (B) rabbit (Rb) antibody or (C) mouse (Ms) antibody directed towards K5. (D) Western blot for detection of ORF45 lytic protein from the same lysates to confirm lytic induction in WT and K5 lines.

Supplemental S4

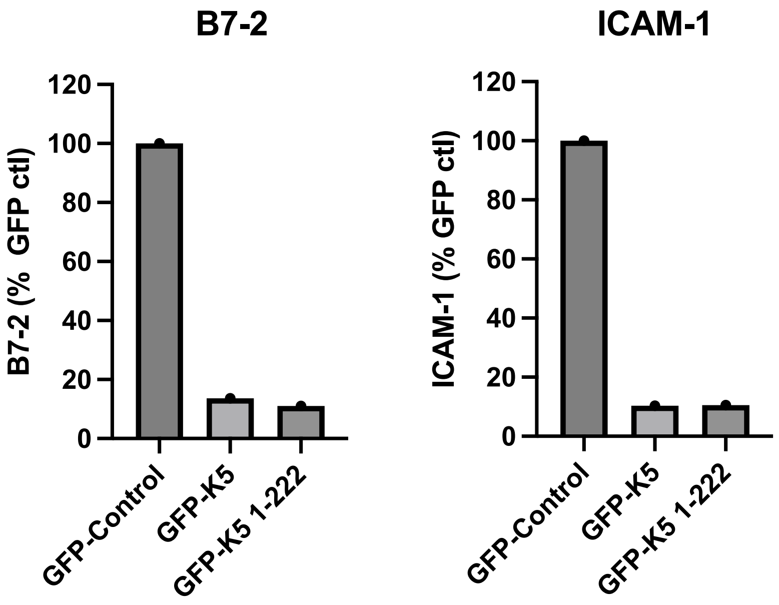

Figure S4: B7-2 and ICAM-1 surface expression in BJAB cells transfected with GFP-control vector, GFP-K5 plasmid, or truncated GFP-K5 (1-222) plasmid. Cells were transfected and then surface expression on live cells was measured (28 h after transfection by FACS.

Supplemental Fig. S5

18S rRNA F	GTAACCCGTTGAACCCCATT
18S rRNA R	CCATCCAATCGGTAGTAGCG
K8.1 F	CCGTCGGTGTGTAGGGATAAAG
K8.1 R	GTCGTTGTAGTGGTGGCAGAAA
ORF6_F	CTGCCATAGGAGGGATGTTTG
ORF6_R	CCATGAGCATTGCTCTGGCT
Myco-16S-F	GTTTGATCCTGGCTCAGGAYDAAC
Myco-16S-R	GAAAGGAGGTRWTCCAYCCSCAC
hu_GAPDH_F	CAGAACATCATCCCTGCCTCTACT
hu_GAPDH_R	GCCGAGCTTCCCGTTCA
K5 primer duplex sequences	5' TAAGCACTTGGCTAACAGTGT 3’
	5' GGCCACAGGTTAAGGCGACT 3'

Fig. S5: Forward and reverse primer sequences used in quantitative PCR analysis.

Full scans for immunoblots

Fig 1A (top)

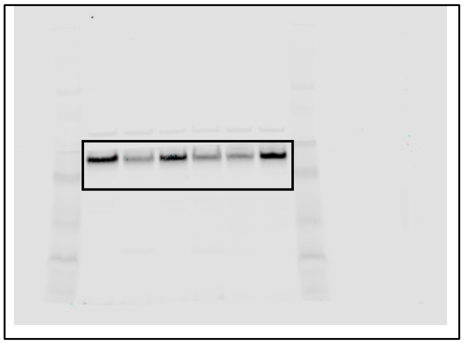

Fig 1A (middle)

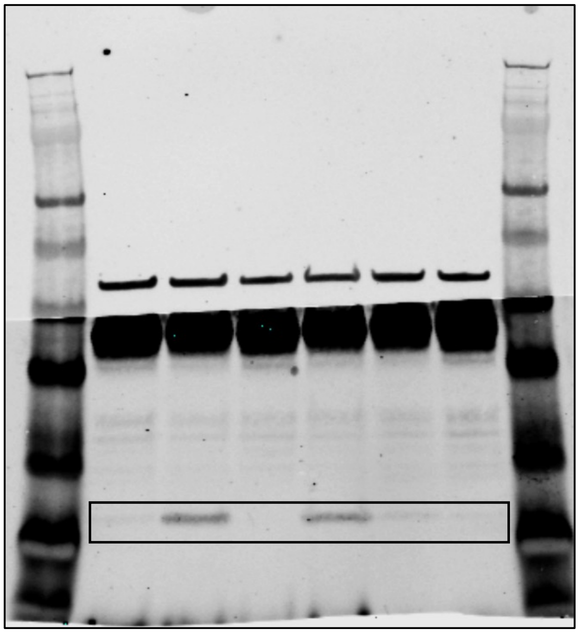

Fig 1A (bottom, actin)

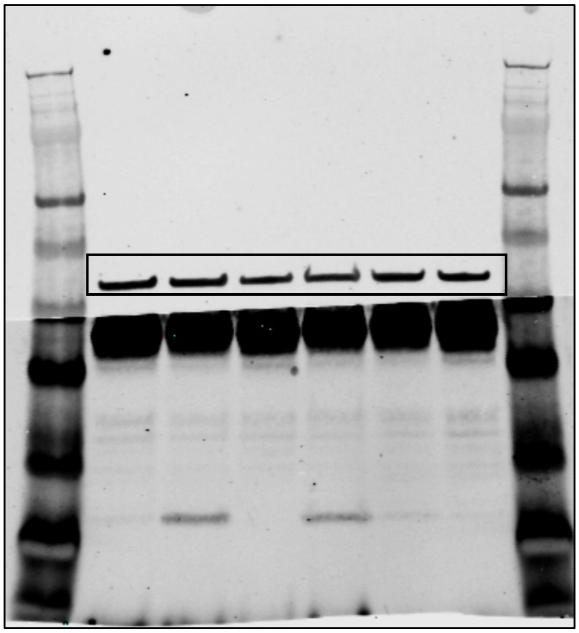

Fig 1B

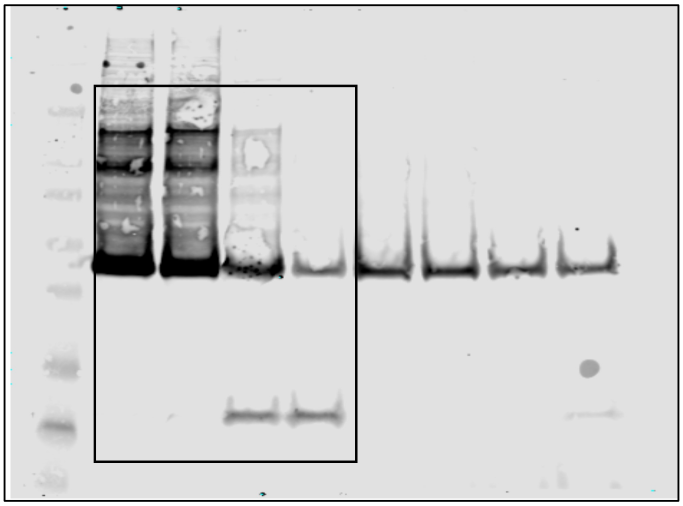

Full scans for immunoblots continued

Fig 2

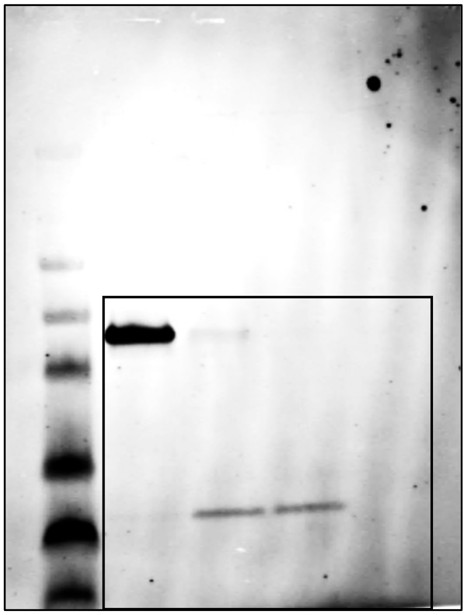

Fig 3A

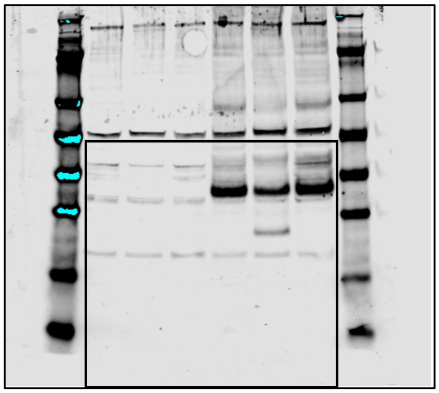

Fig 3B

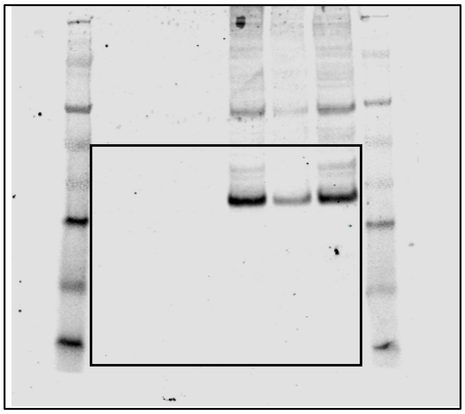

Fig 4A (top)

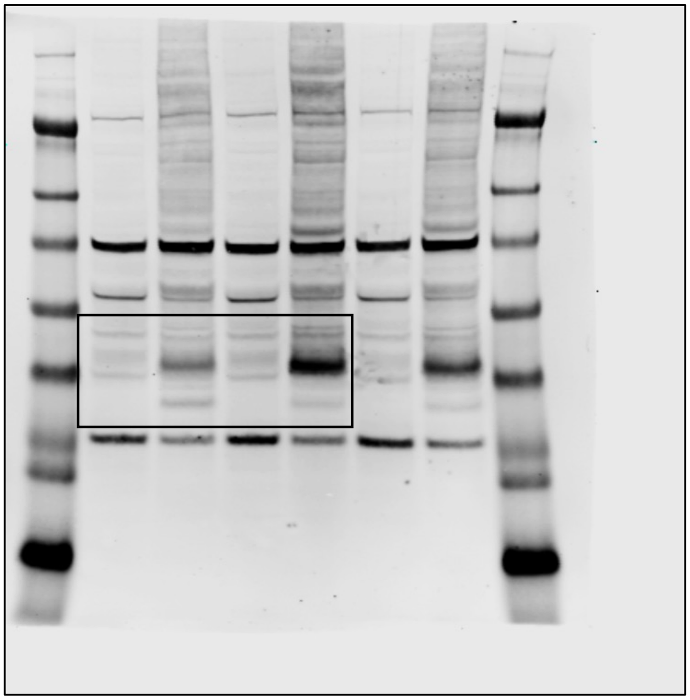

Fig 4A (bottom, actin)

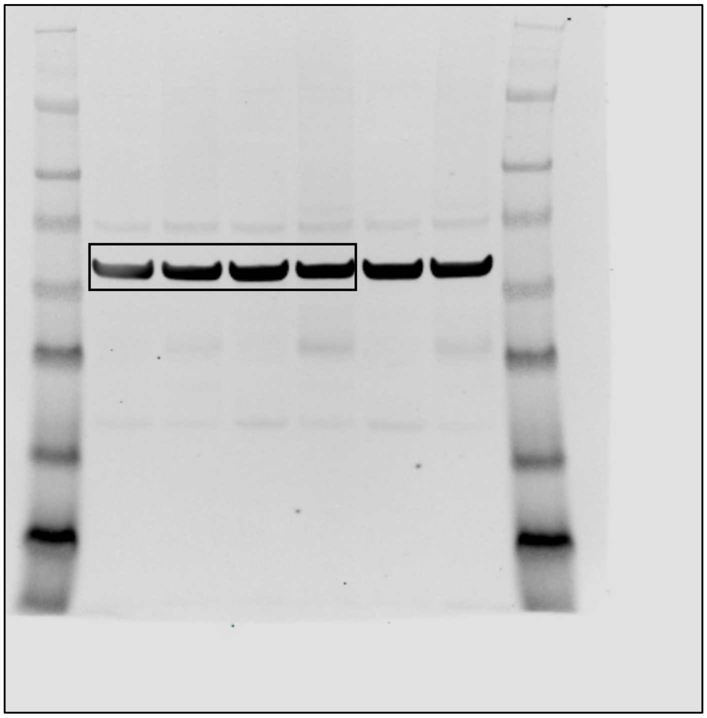

Full scans for immunoblots continued

Fig 4B (top)

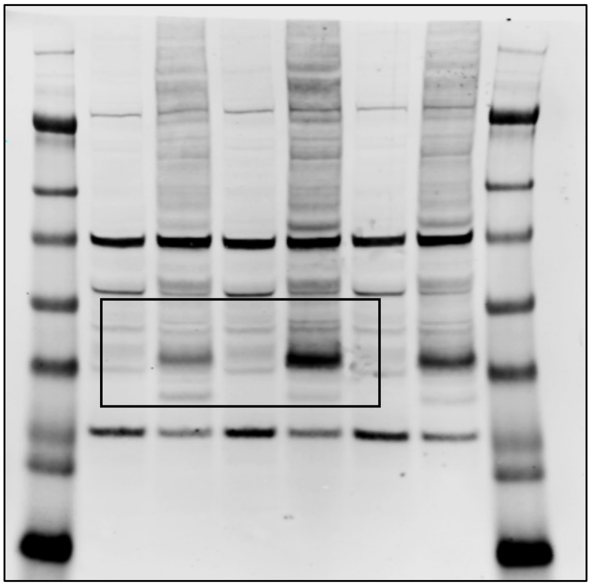

Fig 4B (bottom, actin)

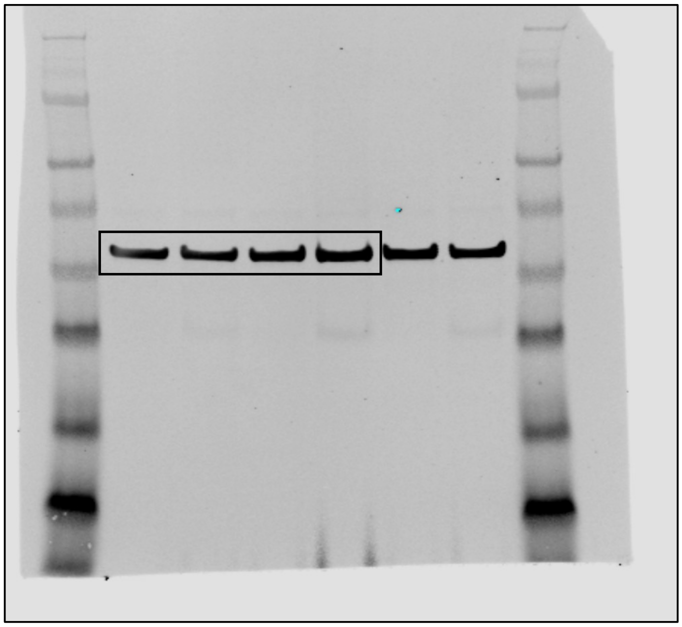

Fig 4C (top)

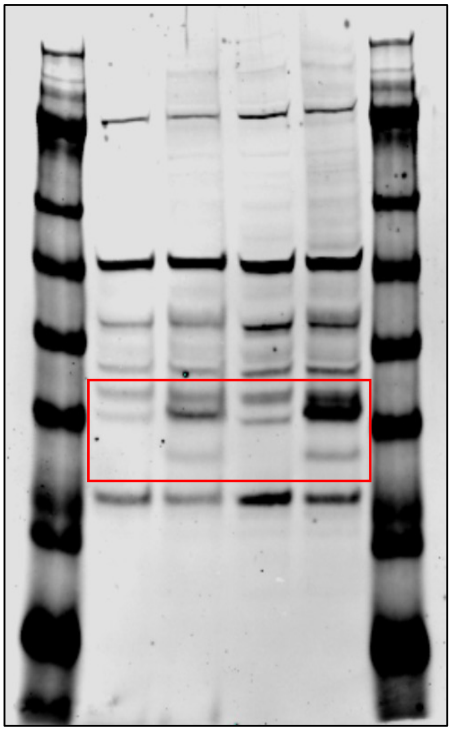

Fig 4C (bottom)

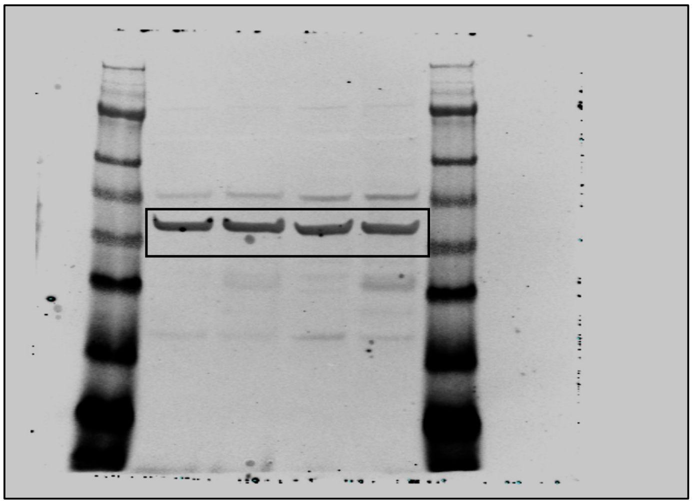

Fig 4D (top)

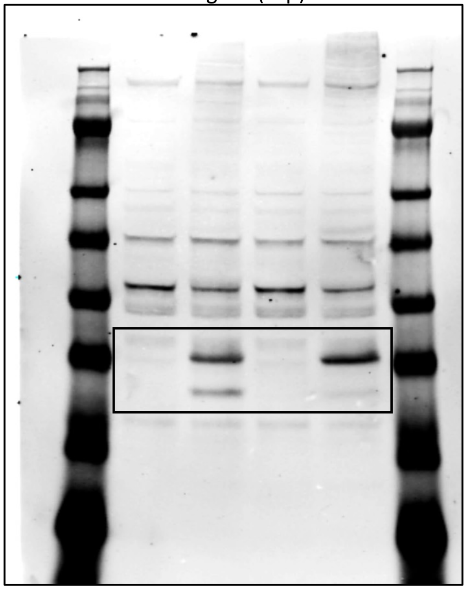

Fig 4D (bottom)

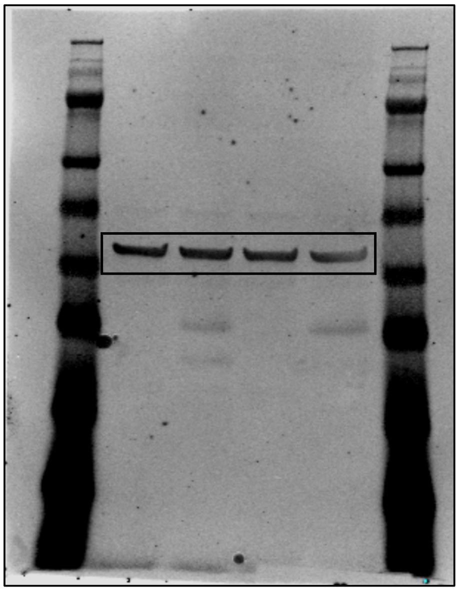

Full scans for immunoblots continued

Fig 4E (top)

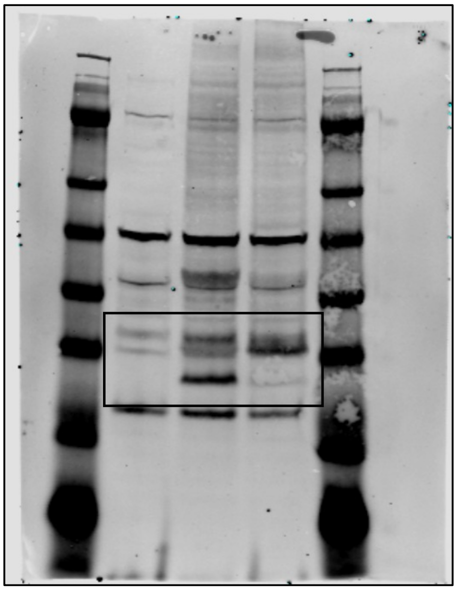

Fig 4E (bottom)

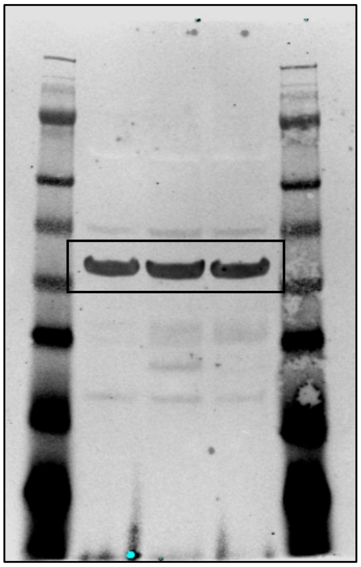

Fig 4F (top)

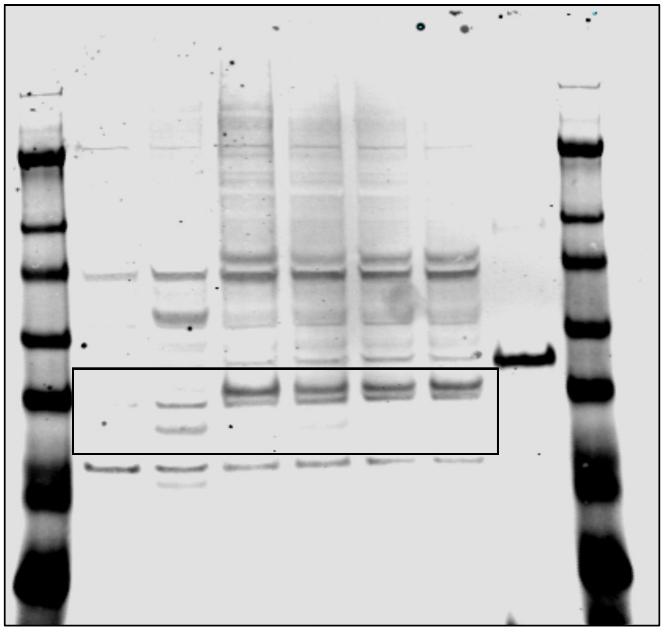

Fig 4F (bottom)

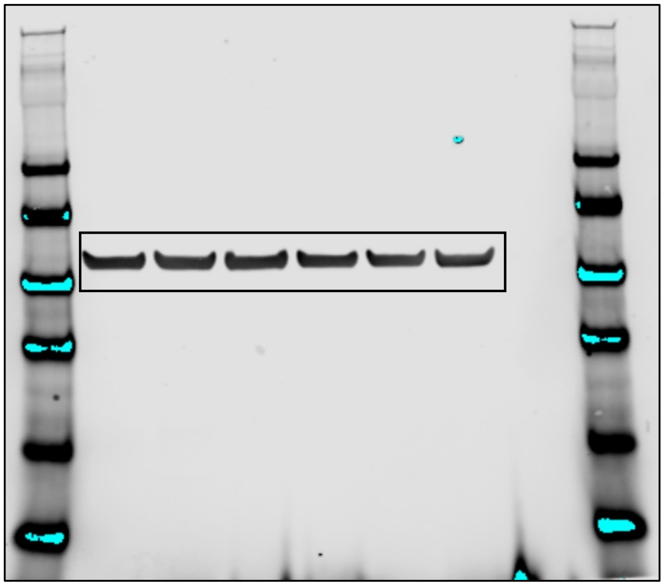

Full scans for immunoblots continued

Fig 5E (top)

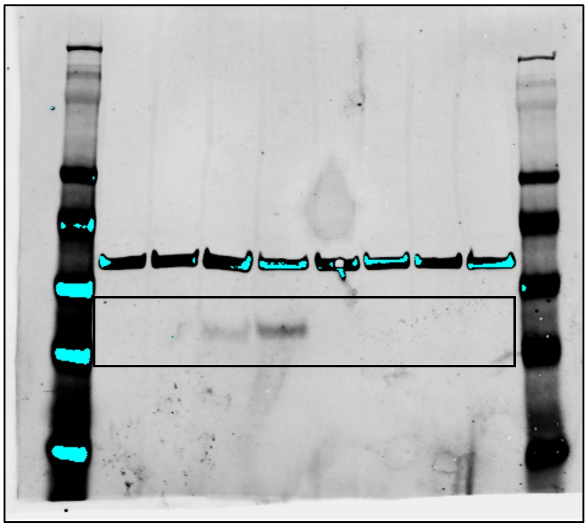

Fig 5E (middle)

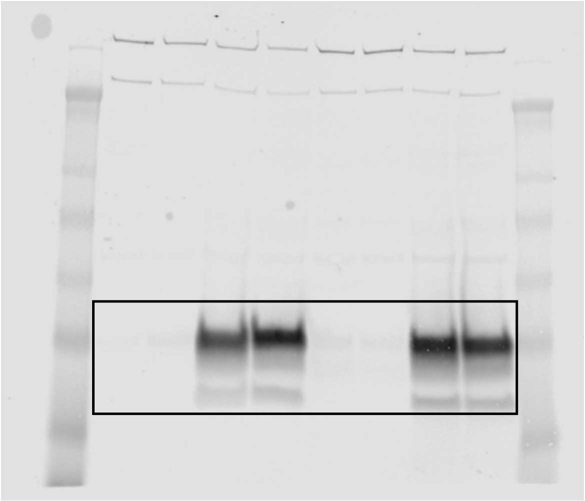

Fig 5E (bottom)

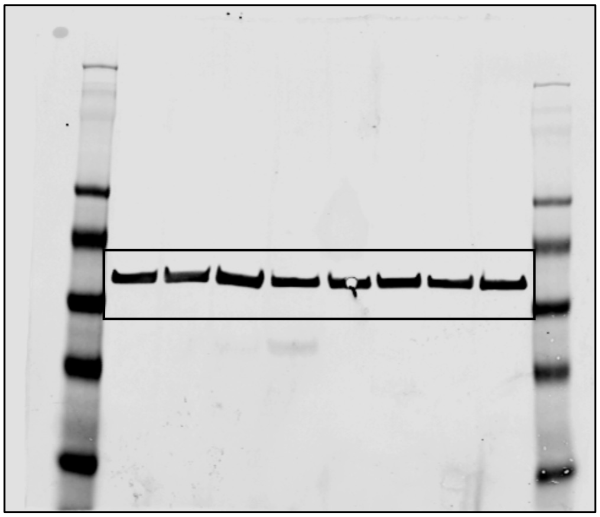

Full scans for immunoblots continued

Fig 6A

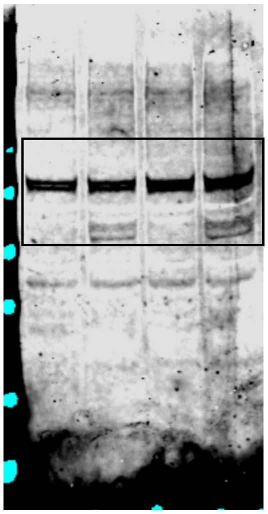

Fig 6B

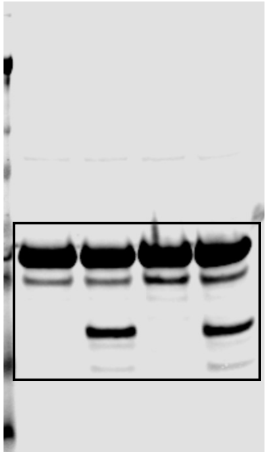

Fig 6C

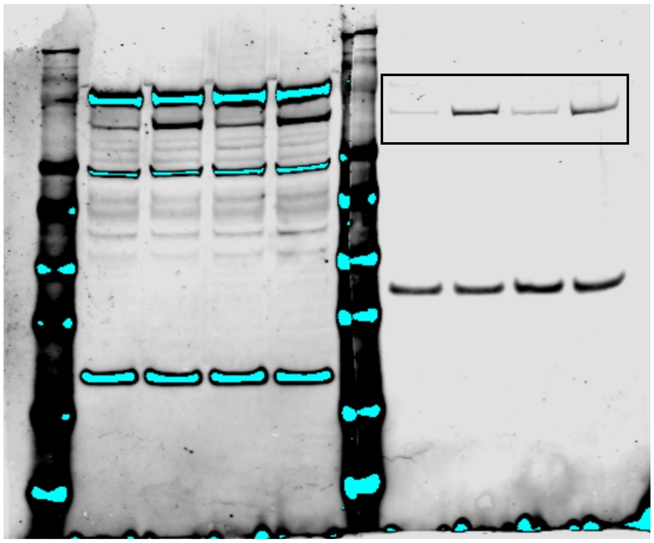

Fig 6D

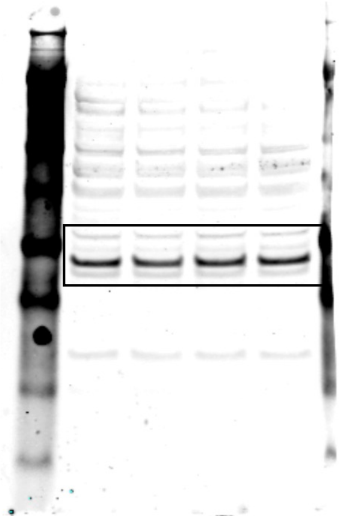

Actin for Fig 6 A-C

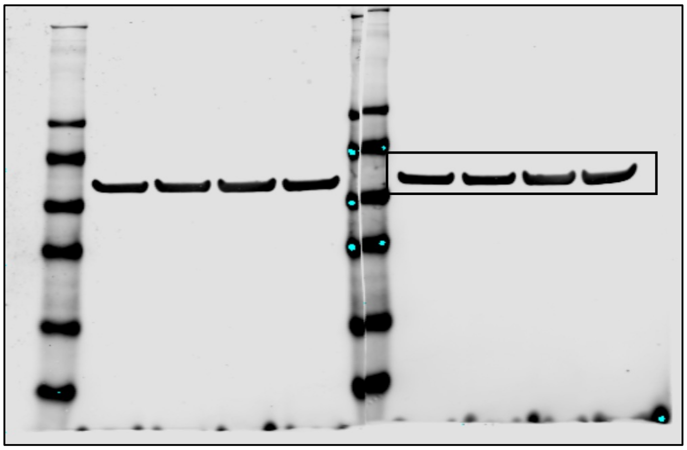

Fig 6E

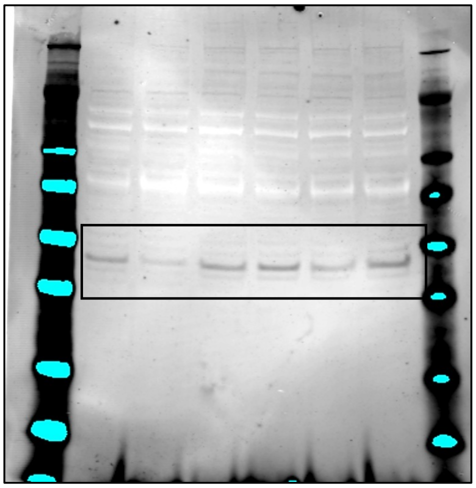

Actin for Fig 6D

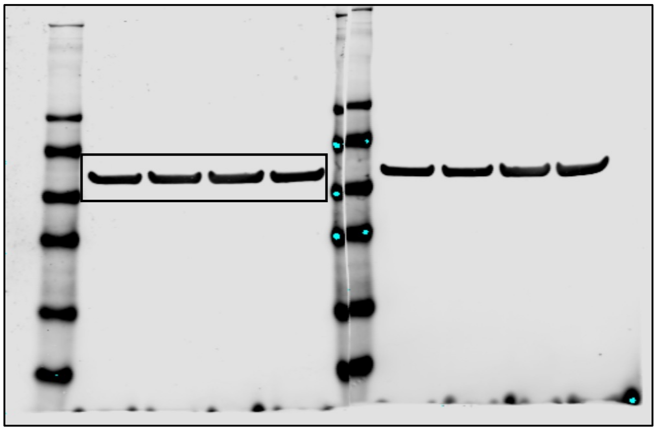

Actin for Fig 6E

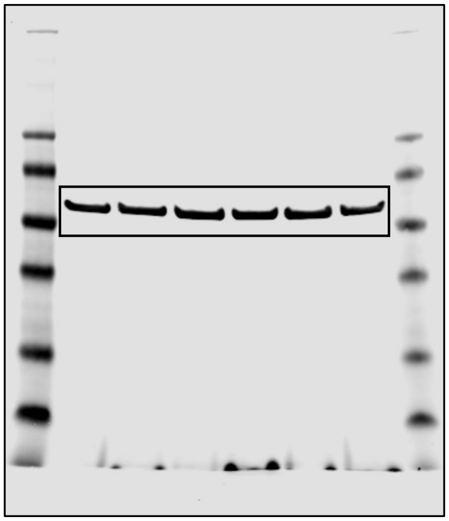

Supplement: Supplemental figures — Figures S1 to S5. [file jvi.00622-25-s0001.pdf]
