# Supplementary material for: Caspase cleavage of Kaposi sarcoma-associated herpesvirus proteins: a role for K5 in preventing caspase-mediated cell death during lytic replication
Source: J Virol. 2025 Aug 29;99(9):e00622-25. doi: 10.1128/jvi.00622-25 (PMC12456141; doi:10.1128/jvi.00622-25)
Supplement: Table S1 — KSHV protein sequences translated from the KSHV DNA sequence from BCBL-1 were analyzed for caspase cleavage sites for caspases 1, 3, 6, 7, and 8 using site prediction (SP). [file jvi.00622-25-s0002.pdf]

Table S1

KSHV Protein	caspase site	SP score	Confirmed?
ORF45*	DEED^{99}*EDEE	2787 (Ex)	No
K10.5/vIRF3	EEVD^{88}*DGAG	2679 (Ex)	No
K5/MIR2*	DEPD^{222}*GGPN	1852 (I,Ex,Inf)	This Study (I, Ex)
ORF57/MTA*	DETD^{33}*APTL	1742 (I,Ex, Inf)	Yes (Ex)
ORF38	VDVD^{18}*GEPL	1649 (Ex)	No
ORF27*	VETD^{97}*AI	1146 (Ex)	No
ORF22*	DESD^{648}*GLQS	984 (I, EX, Inf)	No
ORF25*	VEGD^{346}*KA	926 (Ex)	No
ORF10	VEGD^{208}*PE	863 (Ex)	No
ORF17*	VEKD^{518}*APTP	728 (Ex)	No
ORF73 LANA*	DSVD^{53}*GREC	686 (I, Ex)	Yes (Ex)
ORF63*	TEYD^{529}*ED	518 (Ex)	No
ORF62	VDLD^{208}*ES	504 (Ex)	No
ORF70	VDAD^{174}*AD	487 (Ex)	No
ORF59	ESPD^{332}*SPPL	464 (Inf)	No
ORF67	VESD^{116}*VY	424 (Ex)	No
ORF37*	DTLD^{21}*GLTV	413 (Ex)	No
ORF64*	DESD^{1966}*TASG	392 (Ex)	No
K8*	IEED^{28}*LS	320 (I, Ex)	No
ORF54	GETD^{93}*KD	297 (Ex)	No
ORF50/RTA	DSPD^{408}*NPSS	276 (Inf)	No
K11/vIRF2*	LAPD^{27}*SPRP	274 (Inf)	No
ORF6*	TEED^{1039}*VI	262 (Ex)	No
ORF34	EAVD^{139}*GLCD	256 (Ex)	No
ORF21/TK*	TDDD^{116}*SG	253 (Ex)	No
ORF75*	IEDD^{184}*VI	252 (Ex)	No
ORF71/vFLIP	TDVD*ALMS	214 (Ex)	No
K3/MIR1*	EDED^{5}*VP	183 (Ex)	No
ORF4	SLTD^{495}*SA	180 (I, Ex, Inf)	No
ORF19	LELD^{71}*RL	153 (Ex)	No
ORF20	EVLD^{30}*SSSE	135 (Ex)	No
K14*	TDSD^{178}*GLTV	127 (Ex)	No
ORF74/GPCR	LDDD^{14}*ES	125 (Ex)	No
ORF2	SSLD^{94}*AALG	115 (Ex)	No
ORF49	QELD^{31}*TL	101 (Ex)	No
ORF9	GDTD754*SL	99	No
ORF39	HESD389*SEID	96	No

ORF68	VSED461*VL	96	No
ORF51	ELTD134*AL	85	No
ORF32	VFMD286*DL	84	No
ORF67.5	VSTD70*AVFS	72	No
ORF29	VVGD445*AA	71	No
ORF29b	VVGD109*AA	71	No
ORF23	VVID159*EA	67	No
ORF31	VDRD108*AY	67	No
ORF35	ALVD99*AVAD	66	No
ORF44	TDMD11*EP	64	No
K2	FEKD*LL	59	No
ORF56	LQED*GLER	59	No
ORF55	QEID42*LG	58	No
ORF61	EGED575*AS	58	No
ORF39	VSLD396*TL	55	No
ORF26	AELD175*LY	54	No
ORF43	VVED329*FV	54	No
ORF72	VVED124*FV	54	No
ORF33	VLDD295*GT	53	No
ORF46	DDRD24*LLLA	53	No
ORF42	NEDD232*KQ	50	No
Glyc B	EDLD679*NTID	48	No
ORF30	NEKD15*FE	47	No
ORF7	VLVD667*KK	41	No
ORF28	GMVD15*GSVL	31	No
ORF24	ASPD217*AQ	30	No
ORF60	DSVD5*RFLY	26	No
K1	site not found (snf)	0	NA
K4	snf	0	NA
K4.1	snf	0	NA
K4.2	snf	0	NA
K6	snf	0	NA
K7	snf	0	NA
K9	snf	0	NA
K12a	snf	0	NA
ORF11	snf	0	NA
ORF16/vBcl2	snf	0	NA
ORF18	snf	0	NA
ORF29a	snf	0	NA

ORF36	snf	0	NA
ORF41	snf	0	NA
ORF47	snf	0	NA
ORF52	snf	0	NA
ORF53	snf	0	NA
ORF58	snf	0	NA
ORF65	snf	0	NA
ORF66	snf	0	NA
ORF69	Snf	0	NA

Supplemental Table 1: KSHV protein sequences translated from the KSHV DNA sequence from BCBL-1 (accession number U93872.2) were analyzed for caspase cleavage sites for caspases-1,3,6,7, and 8 using site prediction (SP). The top scoring site for each protein is shown in column 2. Column 3 shows score and class of caspases (initiator caspase-8 (In), inflammatory caspase-1 (Inf), executioner caspases-3,6,7 (Ex)) predicted to cut that site. Column 4 indicates whether the cut site has been experimentally confirmed in this study or in the literature. An * indicates that this protein has one or more other sites scoring over 100; these cut sites are shown in Table 1.
